# Supplementary material for: NCS1 overexpression restored mitochondrial activity and behavioral alterations in a zebrafish model of Wolfram syndrome
Source: Mol Ther Methods Clin Dev. 2022 Oct 7;27:295–308. doi: 10.1016/j.omtm.2022.10.003 (PMC9594121; doi:10.1016/j.omtm.2022.10.003)
Supplement: Document S1. Figures S1–S4 and Tables S1 — –S3 [file mmc1.pdf]

OMTM, Volume *27*

Supplemental information

NCS1 overexpression restored mitochondrial activity and behavioral alterations in a zebrafish model of Wolfram syndrome

Lucie Crouzier, Elodie M. Richard, Camille Diez, Morgane Denus, Amandine Peyrel, Hala Alzaeem, Nicolas Cubedo, Thomas Delaunay, Tangui Maurice, and Benjamin Delprat

Table S1. Two-way ANOVA statistical values for Figure 3D.

gene	*Factor*	*F (DFn, DFd)*	*P value*
sigmar1	Genotype	F (1, 16) = 0,2601	P=0,6170
	Tunicamycin treatment	F (1, 16) = 74,08	P<0,0001 ***
	Interaction	F (1, 16) = 3,502	P=0,0797
bip	Genotype	F (1, 16) = 0,007929	P=0,9302
	Tunicamycin treatment	F (1, 16) = 30,31	P<0,0001 ***
	Interaction	F (1, 16) = 0,0002152	P=0,9885
hsp90b1	Genotype	F (1, 16) = 3,096	P=0,0976
	Tunicamycin treatment	F (1, 16) = 51,73	P<0,0001 ***
	Interaction	F (1, 16) = 3,403	P=0,0837
ire1	Genotype	F (1, 16) = 4,443	P=0,0512
	Tunicamycin treatment	F (1, 16) = 218,2	P<0,0001 ***
	Interaction	F (1, 16) = 5,350	P=0,0344 *
xbp1s	Genotype	F (1, 16) = 0,01421	P=0,9066
	Tunicamycin treatment	F (1, 16) = 31,72	P<0,0001 ***
	Interaction	F (1, 16) = 0,01421	P=0,9066
xbp1us	Genotype	F (1, 16) = 0,3365	P=0,5699
	Tunicamycin treatment	F (1, 16) = 29,26	P<0,0001 ***
	Interaction	F (1, 16) = 0,5956	P=0,4515
perk	Genotype	F (1, 16) = 4,275	P=0,0553
	Tunicamycin treatment	F (1, 16) = 61,18	P<0,0001 ***
	Interaction	F (1, 16) = 3,713	P=0,0720
eif2s1	Genotype	F (1, 13) = 0,002406	P=0,9616
	Tunicamycin treatment	F (1, 13) = 15,71	P=0,0016 **
	Interaction	F (1, 13) = 1,754	P=0,2082
atf4a	Genotype	F (1, 16) = 0,02127	P=0,8859

	Tunicamycin treatment	F (1, 16) = 18,30	P=0,0006 ***
	Interaction	F (1, 16) = 0,01598	P=0,9010
atf4b	Genotype	F (1, 16) = 3,545	P=0,0781
	Tunicamycin treatment	F (1, 16) = 31,66	P<0,0001 ***
	Interaction	F (1, 16) = 0,04306	P=0,8382
atf6	Genotype	F (1, 16) = 4,489	P=0,0501
	Tunicamycin treatment	F (1, 16) = 105,0	P<0,0001 ***
	Interaction	F (1, 16) = 4,620	P=0,0473 *
chop	Genotype	F (1, 11) = 1,601	P=0,2319
	Tunicamycin treatment	F (1, 11) = 27,50	P=0,0003 ***
	Interaction	F (1, 11) = 1,143	P=0,3080

**p < 0.05, **p < 0.01, ***p < 0.001*

Table S2. Two-way ANOVA statistical values for Figure 5.

	Factor	*F (DFn, DFd)*	*P value*
(B) Proteins	Ncs1 OE	F (1, 12) = 24,84	P=0,0003 ***
	Genotype	F (1, 12) = 0,02641	P=0,8736
	Interaction	F (1, 12) = 1,059	P=0,3236
(D) Distance	Ncs1 OE	F (1, 150) = 1,528	P=0,2184
	Genotype	F (1, 150) = 0,01625	P=0,8987
	Interaction	F (1, 150) = 0,6046	P=0,4380
(E) ON	Ncs1 OE	F (1, 150) = 1,708	P=0,1933
	Genotype	F (1, 150) = 2,256	P=0,1352
	Interaction	F (1, 150) = 1,805	P=0,1812
(F) OFF	Ncs1 OE	F (1, 150) = 7,986	P=0,0054 **
	Genotype	F (1, 150) = 7,434	P=0,0072 **
	Interaction	F (1, 150) = 6,419	P=0,0123 *

Abbreviation: Ncs1 OE, Ncs1 over-expression.

**p < 0.05, **p < 0.01, ***p < 0.001*

Table S3. Two-way ANOVA statistical values for Figure 6.

	Factor	*F (DFn, DFd)*	*P value*
(B) Basal respiration	Ncs1 OE	F (1, 120) = 5,548	P=0,0201 *
	Genotype	F (1, 120) = 0,3201	P=0,5726
	Interaction	F (1, 120) = 2,743	P=0,1003
(C) ATP production	Ncs1 OE	F (1, 120) = 1,695	P=0,1954
	Genotype	F (1, 120) = 0,5904	P=0,4438
	Interaction	F (1, 120) = 0,1777	P=0,6741
(D) Maximal respiration	Ncs1 OE	F (1, 120) = 3,387	P=0,0682
	Genotype	F (1, 120) = 6,561	P=0,0117 *
	Interaction	F (1, 120) = 6,391	P=0,0128 *
(E) Proton leak	Ncs1 OE	F (1, 120) = 4,238	P=0,0417 *
	Genotype	F (1, 120) = 0,2717	P=0,6031
	Interaction	F (1, 120) = 6,468	P=0,0123 *
(F) Non mitochondrial respiration	Ncs1 OE	F (1, 120) = 1,012	P=0,3165
	Genotype	F (1, 120) = 1,891	P=0,1716
	Interaction	F (1, 120) = 3,352	P=0,0696

Abbreviation: Ncs1 OE, Ncs1 over-expression.

$p < 0.05$

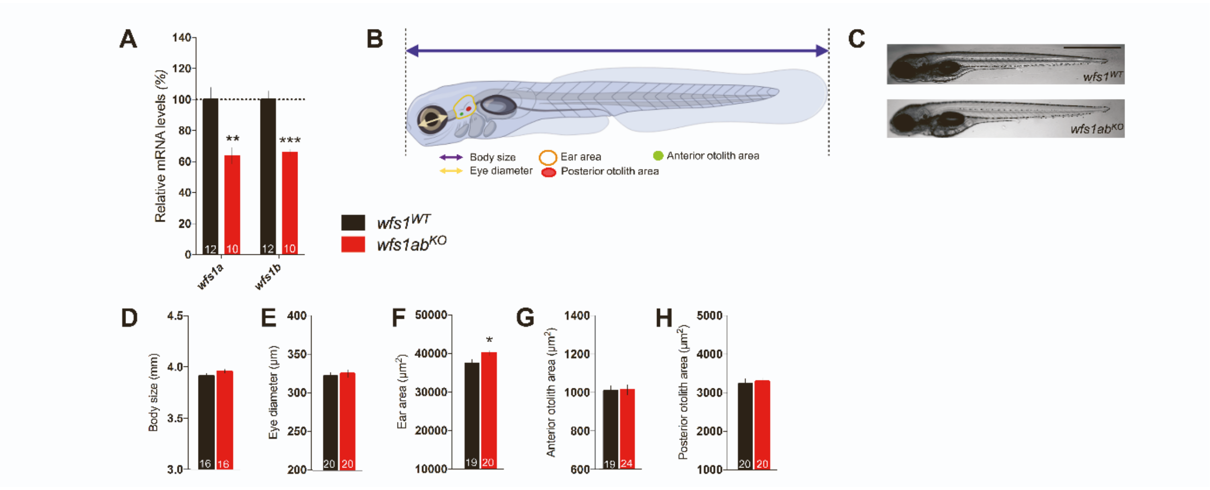

Figure S1. Characterization of *wfs1ab* mutant zebrafish line. (**A**) Relative *wfs1a* and *wfs1b* mRNA levels assessed by qPCR in *wfs1ab* zebrafish line, normalized against *zef1α* reference gene. (**B**) Schematic representation of the different measurements of the larva. (**C**) Representative images of wildtype and homozygous mutant *wfs1ab* larvae. Measurement of (**D**) body size, (**E**) eye diameter, (**F**) ear area, (**G**) anterior and (**H**) posterior otoliths area. Scale bars, 200 µm in D, 10 µm in the insets. Error bars represent mean ± SEM and the number of fish is indicated below the columns. $^{*}p < 0.05$, $^{**}p < 0.01$, $^{***}p < 0.001$; unpaired t-test.

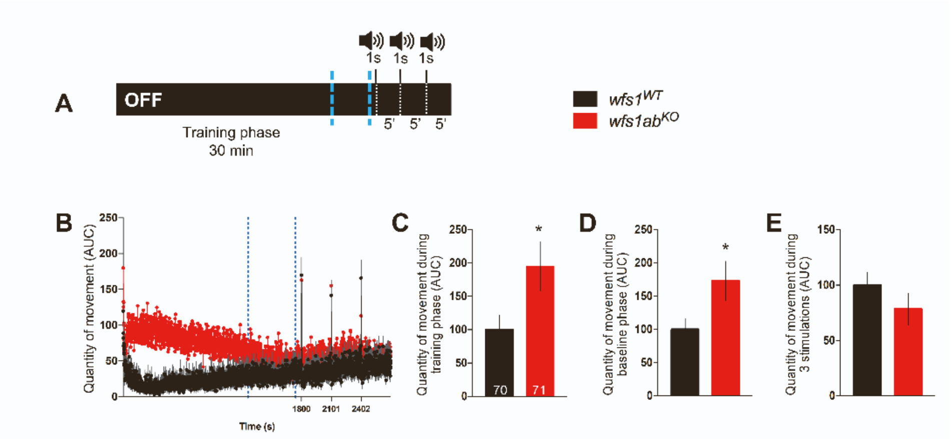

Figure S2. Analysis of the quantity of movement of *wfs1ab* mutant larvae during the noise cycle in the ASR test. (**A**) Sound protocol: the activity is measured for 45 min in a total dark (OFF) condition, with a training phase for 30 min silently, then 3 cycles of white sounds (90 dB) of 1 s each and 5 min interspersed. (**B**) Quantity of movement per second for the *wfs1ab*^{KO} larvae according to the sound protocol. Relative quantity of movement during: (**C**) the training phase [blue dotted lines in (**B**), between 21 and 29 min]; (**D**) the baseline phase, period of 2 min before each sound [the averaged 3 baseline phases]; (**E**) the 3 stimulations [the averaged sounds phases]. Activity was expressed as % of associated controls. Error bars represent ± SEM calculated from three replicas. The number of animals is indicated below the columns. *$p < 0.05$; unpaired t-test.

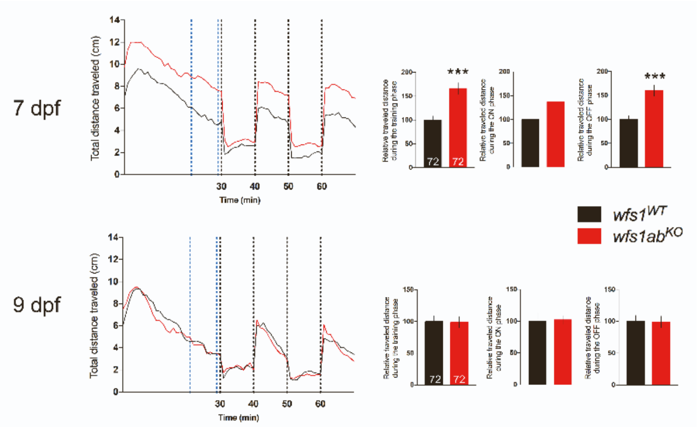

Figure S3. Behavioral analyses of 7-dpf and 9-dpf *wfs1ab*^{KO} zebrafish line. Analysis of the distance traveled by *wfs1ab*^{KO} larvae at 7-dpf (**A**) and 9-dpf (**E**) during the light/dark sequence in the VMR test: training is a light OFF period of 30 min, followed by two light ON and light OFF periods of 10 min each. Relative distance measured during: (**B**, **F**) the training phase over a 10-min period shown by blue dotted lines in (**A**, **E**); (**C**, **G**) the ON phases, averaged for ON1 and ON2; (**D**, **H**) the OFF phases, averaged for OFF1 and OFF2. Data show mean ± SEM, calculated from three replicas. The number of animals is indicated within the columns. ****p < 0.001;* unpaired t-test.

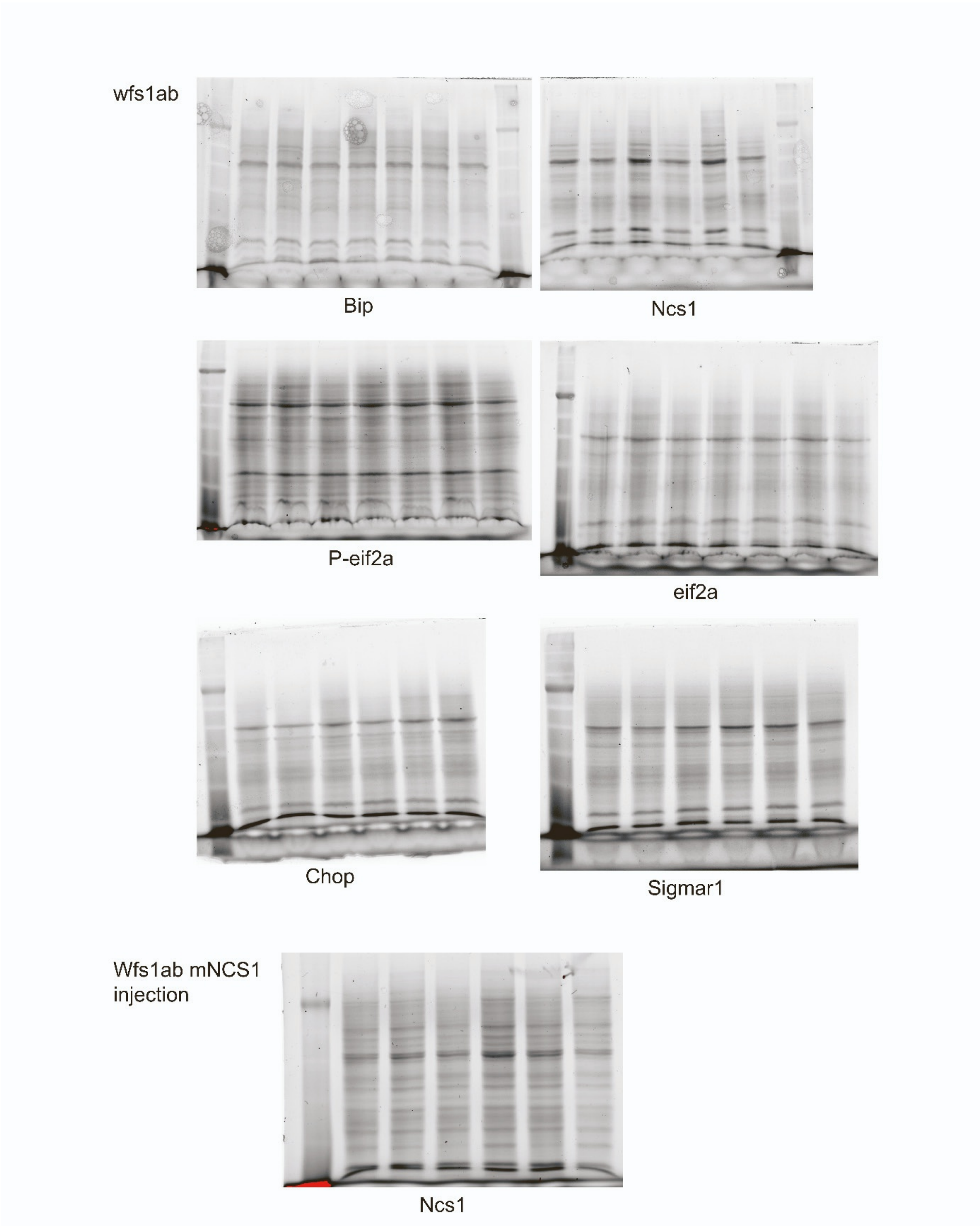

Figure S4. Stain Free for Western blots from Figure 3.
