# Supplementary material for: NCS1 overexpression restored mitochondrial activity and behavioral alterations in a zebrafish model of Wolfram syndrome
Source: Mol Ther Methods Clin Dev. 2022 Oct 7;27:295–308. doi: 10.1016/j.omtm.2022.10.003 (PMC9594121; doi:10.1016/j.omtm.2022.10.003)
Supplement: Document S2. Article plus supplemental information [file mmc2.pdf]

Molecular Therapy
Methods & Clinical Development
Original Article

NCS1 overexpression restored mitochondrial activity and behavioral alterations in a zebrafish model of Wolfram syndrome

Lucie Crouzier,[1,3] Elodie M. Richard,[1,3] Camille Diez,[1] Morgane Denus,[1] Amandine Peyrel,[1] Hala Alzaeem,[1] Nicolas Cubedo,[1] Thomas Delaunay,[2] Tangui Maurice,[1] and Benjamin Delprat[1]

[1]MMDN, Univ Montpellier, EPHE, INSERM, Montpellier, France.; [2]IES, University Montpellier, CNRS, Montpellier, France

Wolfram syndrome (WS) is a rare neurodegenerative disease resulting in deafness, optic atrophy, diabetes, and neurological disorders. Currently, no treatment is available for patients. The mutated gene, *WFS1*, encodes an endoplasmic reticulum (ER) protein, Wolframin. We previously reported that Wolframin regulated the ER-mitochondria Ca^{2+} transfer and mitochondrial activity by protecting NCS1 from degradation in patients' fibroblasts. We relied on a zebrafish model of WS, the *wfs1ab*^{KO} line, to analyze the functional and behavioral impact of NCS1 overexpression as a novel therapeutic strategy. The *wfs1ab*^{KO} line showed an increased locomotion in the visual motor and touch-escape responses. The absence of wfs1 did not impair the cellular unfolded protein response, in basal or tunicamycin-induced ER stress conditions. In contrast, metabolic analysis showed an increase in mitochondrial respiration in *wfs1ab*^{KO} larvae. Interestingly, overexpression of NCS1 using mRNA injection restored the alteration of mitochondrial respiration and hyperlocomotion. Taken together, these data validated the *wfs1ab*^{KO} zebrafish line as a pertinent experimental model of WS and confirmed the therapeutic potential of NCS1. The *wfs1ab*^{KO} line therefore appeared as an efficient model to identify novel therapeutic strategies, such as gene or pharmacological therapies targeting NCS1 that will correct or block WS symptoms.

INTRODUCTION

Rare diseases are defined, according to the European Union, as life-threatening or chronically debilitating diseases which are of such low prevalence, less than 1/2,000 individuals, that they require special combined efforts to be addressed. Most of the time, and because of the limited market these diseases represent, no cure has been developed. Some treatments that only alleviate the symptoms are available to the affected individuals. Among this group of diseases, Wolfram syndrome (WS) is an autosomal recessive genetic disorder characterized by a diabetes mellitus, diabetes insipidus, optic nerve atrophy, hearing loss, and neurodegeneration.[1] The progression of the disease is severe and leads, ultimately, to the premature death of the affected individuals at around 35 years of age with severe neurological disabilities, including bulbar dysfunction and organic brain syndrome.[2] As of today, only symptomatic or substitutive therapies are available to the patients. Therefore, there is an urgent need for a cure that would stop the progression of the pathology.

More than 200 variants in the *WFS1* gene are responsible for WS type 1, which represents 99% of WS cases, while <1% are due to variants in *CISD2* gene.[3–5] *WFS1* encodes a putative 890-amino acid protein, Wolframin, localized into the endoplasmic reticulum (ER) membrane.[6,7] Fonseca et al. have shown in rodent and human cells that WFS1 has a critical function in the regulation of ER stress signaling and prevents secretory cells, such as pancreatic β cells, from dysfunction and premature death caused by hyperactivation of ER stress signaling through its interaction with the transcription factor ATF6α.[8] Since this initial study, accumulating data have established the regulative role of WFS1 on ER stress signaling.[9] One other important function of WFS1 is its role on regulation of mitochondrial calcium transfer through the mitochondria-associated ER membranes (MAMs). These MAMs are involved in a plethora of cellular functions, such as Ca^{2+} homeostasis, ER unfolded protein response (UPR), autophagy, and apoptosis.[10] Interestingly, under physiological conditions, WFS1 interacts with the Ca^{2+} sensor NCS1 in the MAMs and prevents its degradation by forming a complex with the inositol-1,4,5 triphosphate receptor (IP_3R) to activate ER-mitochondria Ca^{2+} transfer.[11] The functionality of the tripartite complex WFS1/NCS1/IP_3R ensures a proper Ca^{2+} transfer from the ER to the mitochondria and the activation of mitochondrial oxidative respiration. In the WS context, we have shown that the decrease of WFS1 in patient fibroblasts is associated with NCS1 downregulation, leading to a decrease in ER-mitochondria Ca^{2+} transfer, triggering *in fine* cell death.[11]

NCS1 is the mammalian ortholog of Frequenin in *Drosophila*, a protein encoded by the *FREQ* gene in humans.[12] NCS1 is one of the EF hand Ca^{2+} sensors that has a high affinity for cellular calcium and

Received 21 February 2022; accepted 4 October 2022;
https://doi.org/10.1016/j.omtm.2022.10.003.

[3]These authors contributed equally

Correspondence: Dr B. Delprat, MMDN, Université de Montpellier, Campus Triolet, CC105, place Eugène Bataillon, 34095, Montpellier cedex 5, France.
E-mail: benjamin.delprat@inserm.fr

This is an open access article under the CC BY license (http://creativecommons.org/licenses/by/4.0/).

thereby functions as a potent transducer of intracellular calcium fluctuations.[13] In mammalian cells, NCS1 promotes exocytosis from dense core vesicles in both neurons and neuroendocrine cells,[14] regulates synaptic transmission,[15] and promotes control of the growth dynamics of nerve endings.[15–17] NCS1 also regulates associative learning and memory in *C. elegans*,[18] and promotes exploration, synaptic plasticity, and rapid acquisition of spatial memory in mice.[19] Very interestingly, NCS1 is highly expressed in retinal ganglion cells, which are highly affected in WS patients.[20] In addition, as highlighted by recent studies, NCS1 plays an essential functional role as a calcium sensor, in pancreatic β cells, key cells in a context of diabetes mellitus.[21,22] In a previous study, we have shown that not only NCS1 knockdown in control fibroblasts recapitulated the metabolic deficits observed in fibroblasts from patients with WS but also that its overexpression in the latter rescued the defective mitochondrial phenotype.[11] A study corroborated our results in different species and cell types, namely rat insulinoma, where *Wfs1* has been knocked out. The overexpression of NCS1 in these cells restored calcium homeostasis deficit and protein kinase B/Akt signaling and, subsequently, cell viability and glucose-stimulated insulin secretion.[22] Considered together, these information conferred to NCS1 a unique therapeutic potential in WS pathology.

Zebrafish (*Danio rerio*) models have proven efficient as animal model of human diseases to find new treatments, particularly in rare genetic diseases.[23] They represent a unique combination of different characteristics, such as short generation time, transparency, small size, and sequenced genome close to the human one, that confer great advantages to this model in therapeutic research.

As most of the genes in the zebrafish genome, *wfs1* is duplicated, both copies *wfs1a* and *wfs1b* sharing, respectively, 52.5% and 52.8% similarities with human *WFS1*. In a previous study, we described two *wfs1* mutant zebrafish lines, specific to each gene, $wfs1a^{C825X}$ and $wfs1b^{W493X}$. These lines mimicked some of the symptoms of WS pathology, including visual, ER stress, and mitochondrial alterations.[24] To fully abolish any Wfs1 functional protein, we intercrossed the two lines mentioned above to create the double-mutant line, $wfs1a^{C825X} \times wfs1b^{W493X}$, termed $wfs1ab^{KO}$ hereafter. The characterization of this line revealed alterations relevant for the study of WS and allowed us to explore the potential therapeutic impact of NCS1 in a WS pathophysiological context.

RESULTS

Characterization of *wfs1ab* mutant zebrafish lines

To validate the double-mutant strain, relative *wfs1a* and *wfs1b* mRNA levels were measured by quantitative PCR (qPCR) in the $wfs1ab^{KO}$ mutant line (Figure S1A). Both genes were significantly downregulated in the double-mutant line, each mutation leading to some mRNA decay, as described previously for each single-mutant line.[24] The protein expression level of *wfs1* could not be assessed, as no commercial antibody specifically labeling the zebrafish protein is available. However, due to the premature stop codons, we predicted that both proteins, when expressed, were not functional.

We assessed the impact of the absence of both Wfs1 proteins on zebrafish larvae morphology using different parameters (detailed in Figure S1B). The fish were measured at 5 dpf using a binocular loupe and analyzed using the Fiji software (Figures S1C–S1H). Overall, the mutations did not impact the grossly observable morphology of the larvae, with the exception of an increased ear area (Figure S1F) in $wfs1ab^{KO}$ fish compared with the controls.

Downregulation of Wfs1 impact visual and locomotor functions of $wfs1ab^{KO}$ zebrafish

The visual motor response (VMR) assay measured the locomotor response of the larvae induced after visual stimulation. Experimentally, larvae at 5 dpf were placed individually in each well of a 96-well plate and their individual activity was measured by videotracking. A training phase of 30 min in the dark (light OFF) allowed the larvae to acclimate to their new environment, followed by two 10-min duration light ON (100% light) and light OFF phases, with a 10-min intertrial time interval. The locomotion profiles are shown in Figure 1A. The locomotion of the $wfs1ab^{KO}$ larvae increased drastically compared with $wfs1ab^{WT}$ controls during the training phase (Figure 1B), ON (Figure 1C), and OFF periods (Figure 1D), highlighting a hyperlocomotor response in $wfs1ab^{KO}$ larvae. The hyperlocomotion is still present at 7 dpf but disappeared at 9 dpf (Figures S3A–S3H).

The visual acuity of the mutant zebrafish was measured using the optokinetic response (OKR). Movements of larva eyes were provoked by rotating black-and-white strips retroprojected on a fixed cylinder and videotracked (Figure 1E). The number of eye saccades within 2-min time intervals, reflecting the visual acuity of the immobilized 5-dpf larva, was not altered for $wfs1ab^{KO}$ larvae compared with $wfs1ab^{WT}$ controls (Figure 1F).

The reflex motor response was also measured, using the touch-evoked escape behavior (Figure 1G). After stimulus, the $wfs1ab^{KO}$ larvae showed a significant increase of the traveled distance (Figure 1H), suggesting a motor alteration that could be related to the increased locomotor response previously observed in the VMR assay.

We also assessed the acoustic startle response (ASR) induced by a repetitive 1-s duration noise (Figure S2) and measured using the quantity of movement (Figure S2A). The quantity of movement during both the training (Figure S2B) and baseline (Figure S2C) phases was increased for $wfs1ab^{KO}$ larvae compared with $wfs1ab^{WT}$ controls. The *wfs1* mutations had, however, no impact on the quantity of movement after stimulation (Figure S2D).

Absence of functional Wfs1 disrupted eye development of zebrafish larvae

As the larvae exhibited visual alterations in the VMR assay, an immunohistochemical analysis of the developing eye was performed at 5 dpf in the $wfs1ab^{KO}$ mutant line (Figure 2). Quantification of cell numbers from immunostained cryosections of the whole retina showed a non-significant decrease of the density of retinal ganglion cells in $wfs1ab^{KO}$

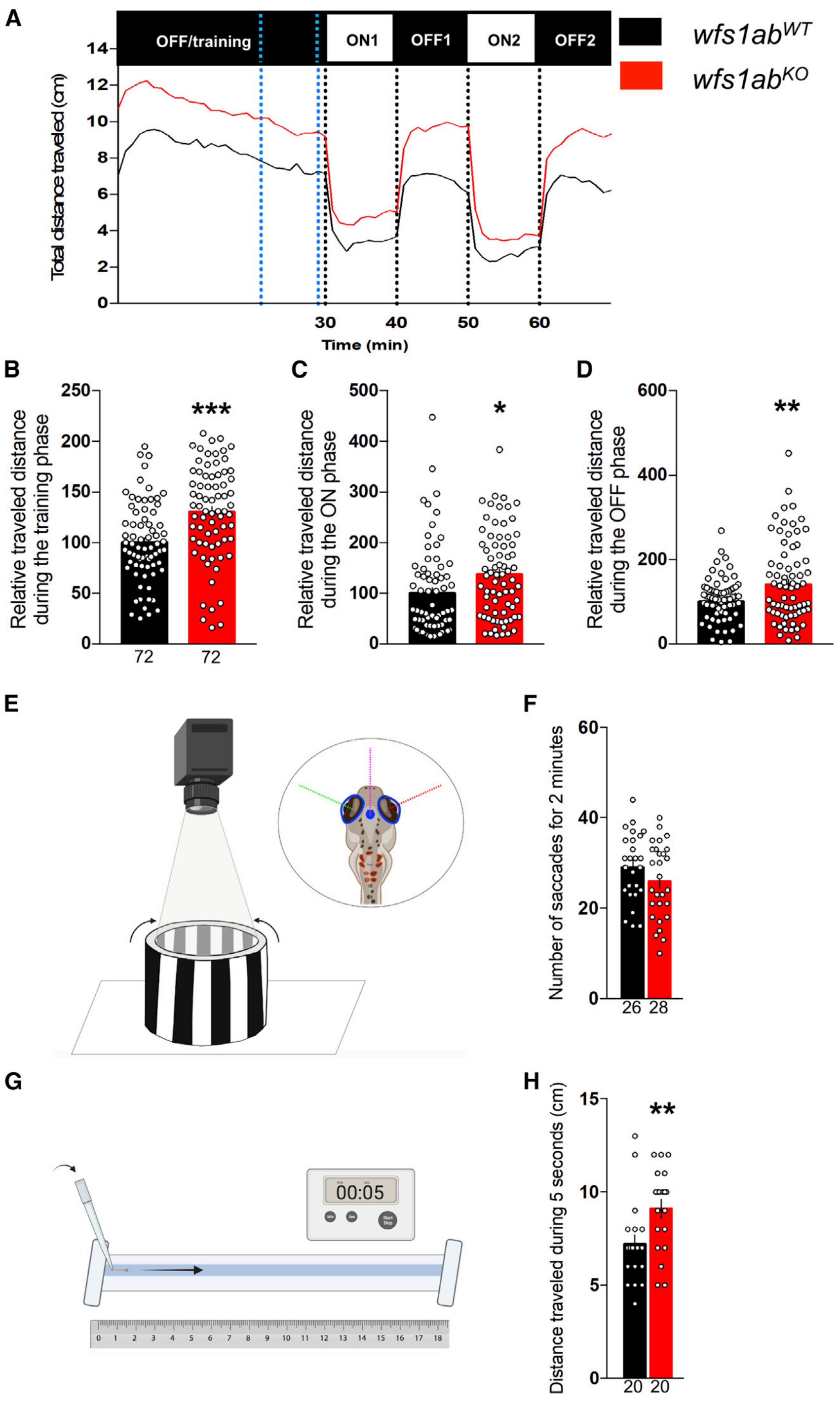

Figure 1. Behavioral analyses of 5 dpf *wfs1ab*^{KO} zebrafish line

(A) Analysis of the relative distance traveled by 5 dpf *wfs1ab*^{KO} larvae during the light/dark sequence in the VMR test: training is a light OFF period of 30 min, followed by two light ON and light OFF periods of 10 min each. (B) The training phase over a 10-min period shown by blue dotted lines in (A); (C) the ON phases, averaged for ON1 and ON2; (D) the OFF phases, averaged for OFF1 and OFF2. (E) Illustration of the OKR assay. Four larvae are immobilized in a Petri dish and placed in an arena with rolling black-and-white strips. (F) Number of saccades within 2 min. (G) Illustration of the touch-escape response test. The tail of the larva is touched with a tip and (H) the traveled distance in the rail is measured for 5 s, repeated three times and averaged. Relative distances were expressed as percent of controls. Data show mean ± SEM, calculated from three replicas. The number of animals is indicated within the columns, n = 72 animals per genotype. $^*p < 0.05$, $^{**}p < 0.01$, $^{***}p < 0.0001$; unpaired t test.

compared with *wfs1ab*^{WT} controls (Figures 2A and 2B), without alteration of the thickness of the ganglion cells layer (Figure 2C). The density of red and green cones, labeled with Zpr-1 antibody (Figures 2D and 2E), as well as the density of rods, labeled with Rho4d2 antibody (Figures 2F and 2G), were decreased in *wfs1ab*^{KO} larvae. The level of glucose did not change in *wfs1ab*^{KO} larvae (Figure 2I).

ER stress response in *wfs1ab*^{KO} zebrafish

The expression level of genes involved in the regulation cascade of the ER stress was analyzed by qPCR in the *wfs1ab*^{KO} mutant zebrafish at 5 dpf (Figure 3A). No alteration of the expression level of UPR pathway inducers (*sigmar1*, *bip*, *hsp90b1*), primary effectors of the UPR pathway (*ire1*, *atf6*, *xbp1s*, *xbp1us*) and secondary effectors (*perk*, *eif2s1*, *atf4α*, *atf4β*, *chop*) was noted, with the notable exception of *eif2s1*, slightly but significantly decreased in *wfs1ab*^{KO} larvae compared with *wfs1ab*^{WT} controls (Figure 3A). Western blot analyses were carried out, only with a handful of antibodies showing a specific labeling in zebrafish tissues. We observed that, out of all the ER stress-related proteins tested, only Chop was significantly downregulated in *wfs1ab*^{KO} larvae compared with *wfs1ab*^{WT} controls (Figures 3B and 3C).

Larvae were treated with tunicamycin, 2 μg/mL for 24 h, to analyze the UPR in condition of ER stress (Figures 3D and 3E). The mRNA level of all genes was significantly increased in the *wfs1ab*^{KO} larvae and *wfs1ab*^{WT} controls treated after tunicamycin, with the notable exception of *eif2s1* in control larvae. The genotype had a mild impact on the gene's response to tunicamycin. Induction of *hsp90b1* and, to a lesser extent, *perk* tended to show a milder increase while *eif2s1* expression was significantly increased in ER stress conditions in *wfs1ab*^{KO} larvae (Figures 3D and 3E). These data suggested that Wfs1 inactivation in zebrafish had only a mild impact, limited to some markers of the

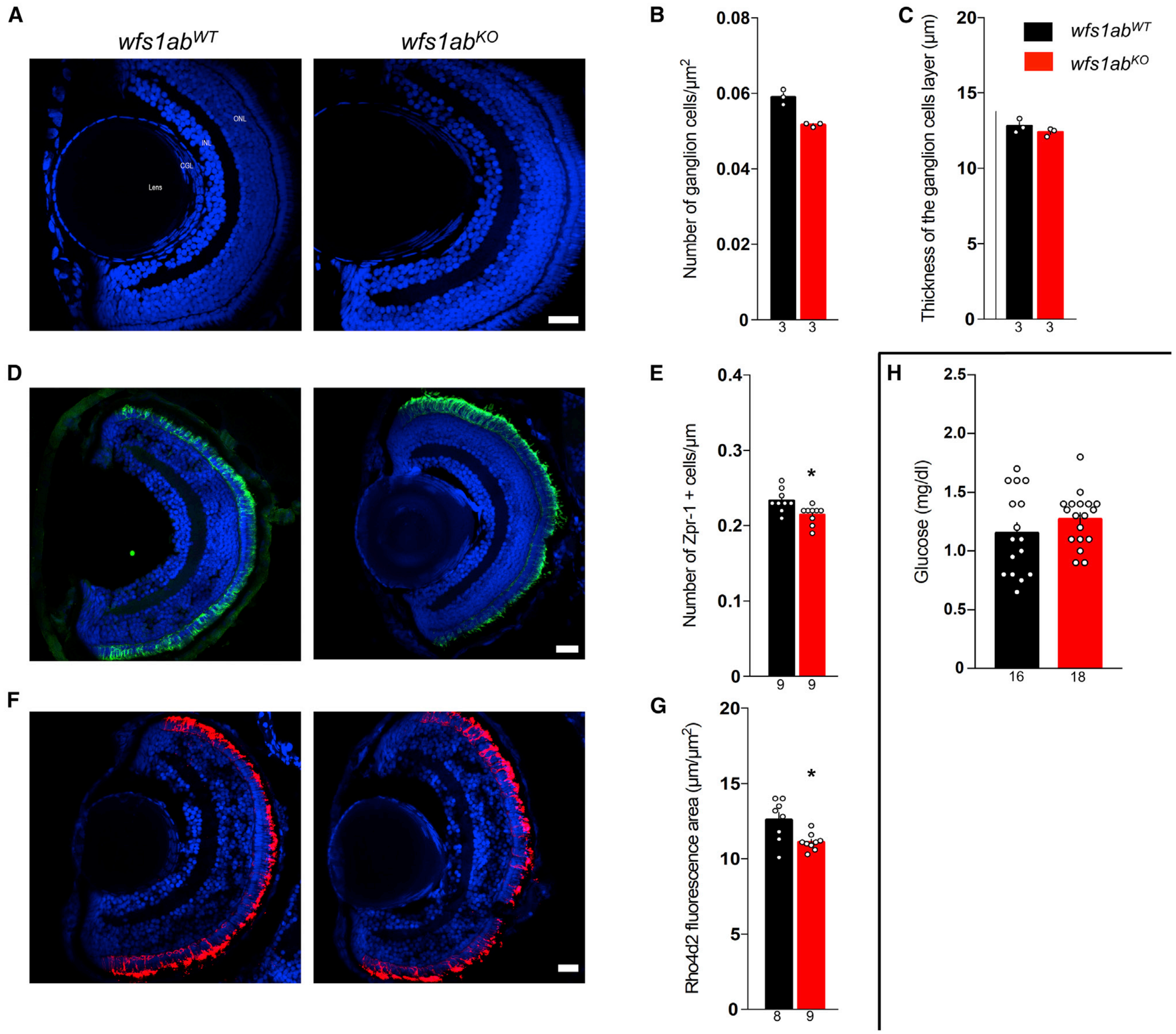

Figure 2. Morphological characterization of the retina and glycemia analysis of the *wfs1ab*^{KO} zebrafish line

(A) Typical micrographs of the retina, (B) quantification of the number of ganglion cells, and (C) quantification of the thickness of the associated layer. Confocal images were obtained from sections from *wfs1ab*^{WT} and *wfs1ab*^{KO} zebrafish retina, showing cell nuclei labeled with 4′,6-diamidino-2-phenylindole (DAPI) (blue). (D and E) (D) Typical micrographs of the cones (green) and (E) quantification of photoreceptor cells (red and green cones) labeled with Zpr-1 antibody. (F and G) (F) Typical micrographs of the rods (red) and (G) quantification of rods labeled with Rho4d2 antibody. (H) Pancreas characterization with measurement of blood glucose on whole larvae. GCL, ganglion cell layer; INL, inner nuclear layer; ONL, outer nuclear layer. Scale bars, 50 µm in (A, D, and F). The number of animals is indicated in the columns. *$p < 0.05$; unpaired t test.

PERK-dependent pathway, while IRE1- and ATF6-dependent pathways were unaffected.

Mitochondrial functions are altered in *wfs1ab*^{KO} zebrafish

Mitochondrial oxidative respiration rates were measured *in vivo* in 5-dpf larvae using the Seahorse XFe24 analyzer to determine the consequences of Wfs1 invalidation on mitochondrial functionality (Figure 4). The oxygen consumption rate (OCR) (Figure 4A) was measured before and after the addition of: (1) the ATP synthase inhibitor oligomycin, to measure the ATP production-related OCR; (2) the uncoupling agent FCCP, to measure the maximal OCR; and (3) antimycin A and rotenone, to measure proton leak and non-mitochondrial OCR (Figure 4A). The *wfs1ab*^{KO} line showed a significant increase in basal respiration (Figure 4B), ATP production-related OCR (Figure 4C), and maximal respiration (Figure 4D) compared with control larvae. No difference was found in proton leak (Figure 4E) or non-mitochondrial respiration (Figure 4F).

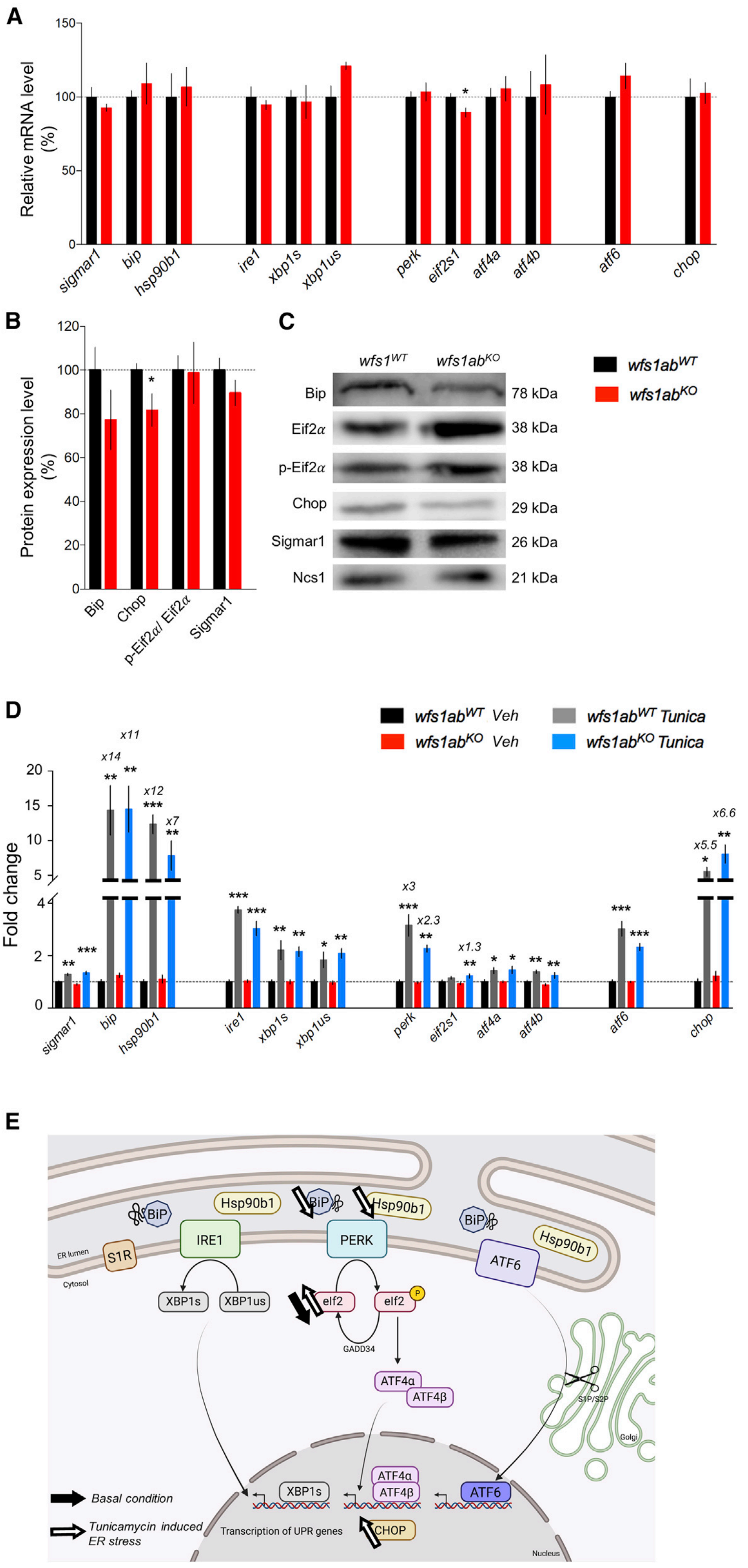

Figure 3. ER stress response in 5 dpf *wfs1ab*^{KO} zebrafish larvae under physiological and ER stress conditions after tunicamycin treatment

(A) mRNA levels of ER stress markers were analyzed by qPCR and (B) protein levels by western blot in *wfs1ab*^{WT} and *wfs1ab*^{KO} larvae. (C) Pictures of typical blots (completely stain free) are shown in Figure S4. (D) Larvae were exposed to vehicle DMSO solution (Veh) or tunicamycin 2 μg/mL (Tunica) for 24 h. *zeif2α* and stain-free were used as a loading control in qPCR and western blot analyses, respectively. The relative expression levels of mRNA after tunicamycin treatment are indicated as the fold change from the respective *wfs1ab*^{WT}/DMSO or *wfs1ab*^{KO}/DMSO control group, when higher than 2-fold. Data are expressed as mean ± SEM, n = 5–9 in each group. *p < 0.05 versus *wfs1ab*^{WT} in (A and B). *p < 0.05, **p < 0.01, ***p < 0.001 versus DMSO treatment in (A and B) and Tukey's multiple comparison test in (D).

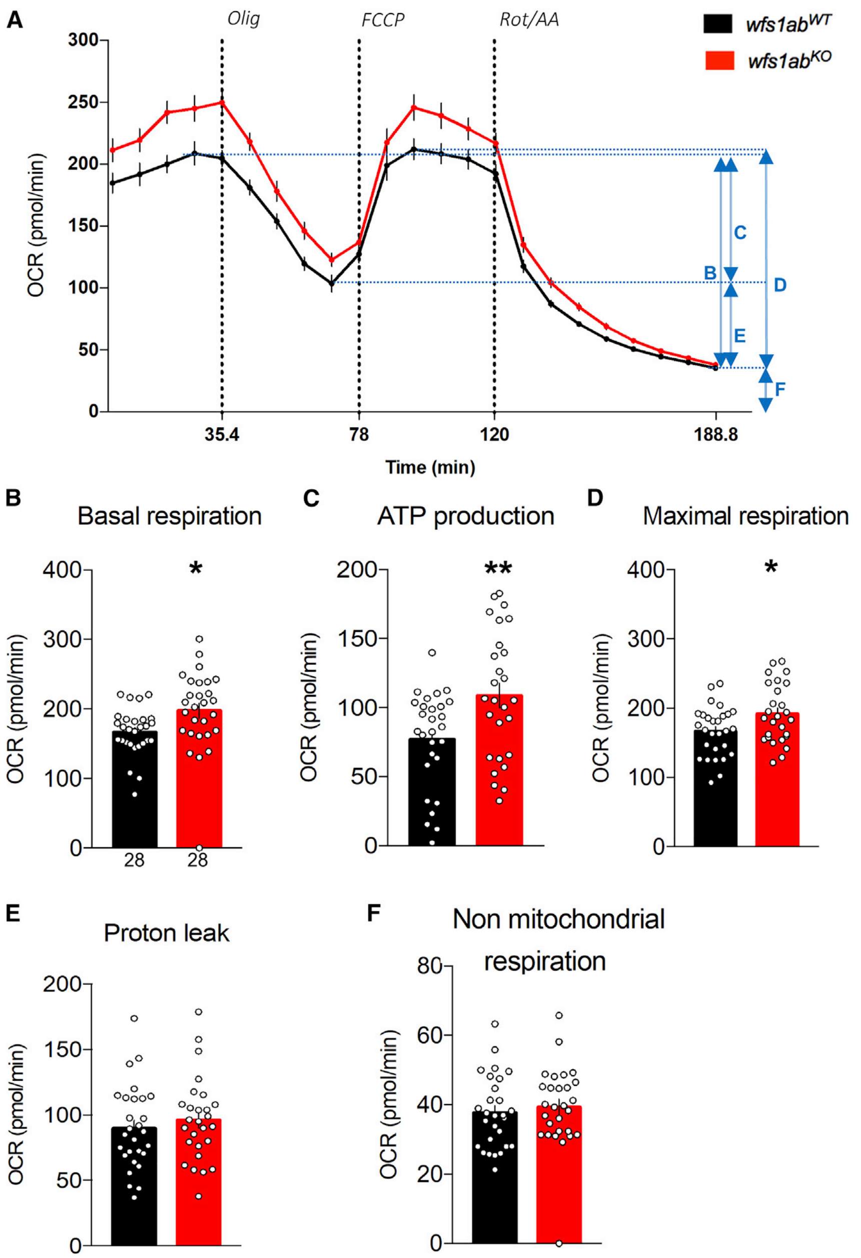

Figure 4. Analysis of mitochondrial respiration in 5 dpf *wfs1*^{KO} zebrafish larvae

(A) Oxygen consumption rate (OCR) profiles of *wfs1ab*^{WT} and *wfs1ab*^{KO} zebrafish larvae at 5 dpf during the assay and calculated, (B) basal respiration rate, (C) ATP production-related OCR, (D) maximal respiration, (E) proton leak, and (F) non-mitochondrial respiration. Data are mean from triplicates and show mean ± SEM from n = 28 animals per genotype. Olig, oligomycin; FCCP, cyanide-p-trifluoromethoxyphenylhydrazone; Rot/AA, rotenone + antimycin A. $^*p < 0.05$, $^{**}p < 0.01$ versus *wfs1ab*^{WT}, unpaired t test.

Overexpression of Ncs1 restored the locomotion and mitochondrial alterations of the *wfs1ab*^{KO} zebrafish

We previously reported that, in WS patient fibroblasts, the overexpression of NCS1 was able to restore all the key physiological deficits responsible of the severity of the disease.[11] Therefore, we examined the impact of NCS1 overexpression in *wfs1ab*^{WT} and *wfs1ab*^{KO} zebrafish lines. mCherry or *Ncs1* RNAs were injected into the eggs and Ncs1 expression was assessed by western blot analysis at 5 dpf. A significant 2-fold overexpression of the protein level was measured in both lines (Figures 5A and 5B). Once the overexpression was validated, we analyzed the motor behavior functions of the Ncs1- or mcherry RNA-injected lines using the VMR assay (Figure 5C). The locomotion profiles were measured (Figures 5D–5F). No significant difference in the distance traveled during the light alternation sequences was measured during the training or ON phases (Figures 5D and 5E), but mCherry RNA-injected *wfs1ab*^{KO} larvae showed a significantly higher response during the OFF phase compared with *wfs1*^{WT} larvae, recapitulating the phenotype observed in non-injected fish. On the other hand, Ncs1 overexpression completely prevented the hyperlocomotion observed in *wfs1ab*^{KO} larvae (Figure 5F).

We also measured the impact of Ncs1 overexpression on mitochondrial function. The OCR profiles were analyzed using the Seahorse analyzer as described previously (Figure 6A). The mcherry RNA-injected *wfs1ab*^{KO} larvae exhibited statistically significant increases of basal respiration (Figure 6B), maximal respiration (Figure 6D), and a trend for ATP production-related OCR ($p = 0.237$; Figure 6C), compared with controls, confirming the previously observed effects (Figure 4). No significant impact of the absence of Wfs1 on proton leak and non-mitochondrial OCR were measured (Figures 6E and 6F).

The injection of Ncs1 non-significantly prevented the increase in basal respiration (Figure 6B) and significantly prevented the increase in maximal respiration (Figure 6D) down to the levels observed in the control *wfs1ab*^{WT} line. These observations confirmed *in vivo* the essential functional role played by Ncs1 in a WS pathophysiological context.

DISCUSSION

The aim of the present study was to characterize a zebrafish model of WS, with non-functional Wfs1a nor Wfs1b proteins, and to challenge the hypothesis that an overexpression of Ncs1 *in vivo* would restore most of the observed behavioral and cellular alterations shown by the mutant line. Contrarily to single mutation models published

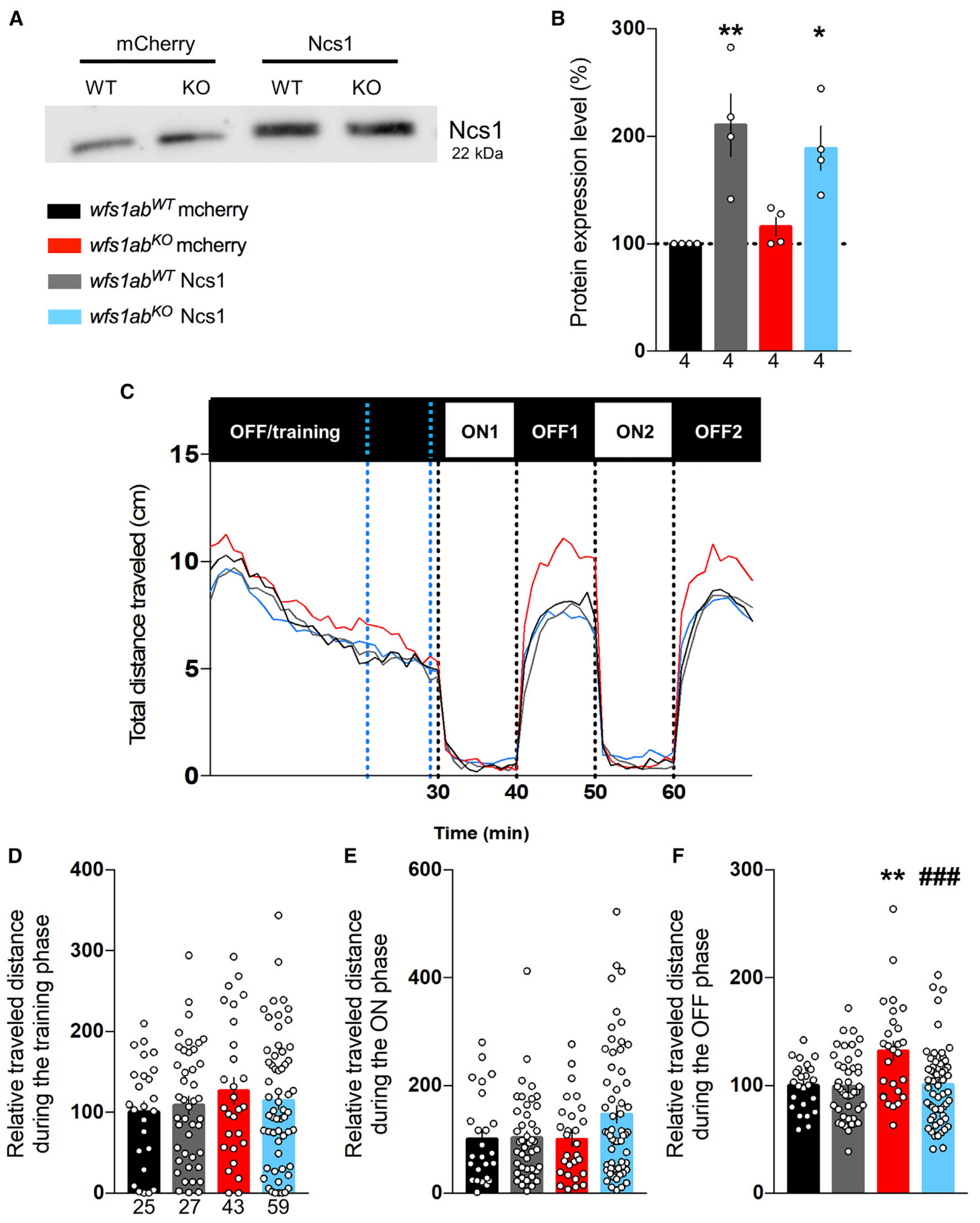

Figure 5. Ncs1 overexpression restores hyperlocomotion in the VMR assay

(A and B) The Ncs1 protein level was measured by western blot in mCherry or *Ncs1* RNA-injected *wfs1ab*^{WT} and *wfs1ab*^{KO} zebrafish larvae at 5 dpf. (C) Analysis of the distance traveled by mCherry- or *Ncs1* RNA-injected *wfs1ab*^{WT} and *wfs1ab*^{KO} larvae, during the light/dark sequence in the VMR assay. Relative distance measured during: (D) the training phase over a 10-min period shown by blue dotted lines in (C); (E) the ON phases, averaged for ON1 and ON2; (F) the OFF phases, averaged for OFF1 and OFF2. Data are mean ± SEM calculated from the number of animals indicated within the columns in (B), n = 4 animals per genotype and (D) for (C–F), n = 25 animals *wfs1ab*^{WT} mCherry, n = 27 animals *wfs1ab*^{KO} mCherry, n = 43 animals *wfs1ab*^{WT} Ncs1, n = 59 animals *wfs1ab*^{KO} Ncs1. *p < 0.05, **p < 0.01 versus *wfs1ab*^{WT}, $^{\#\#}$p < 0.01 versus mCherry RNA treatment; Tukey's multiple comparison test.

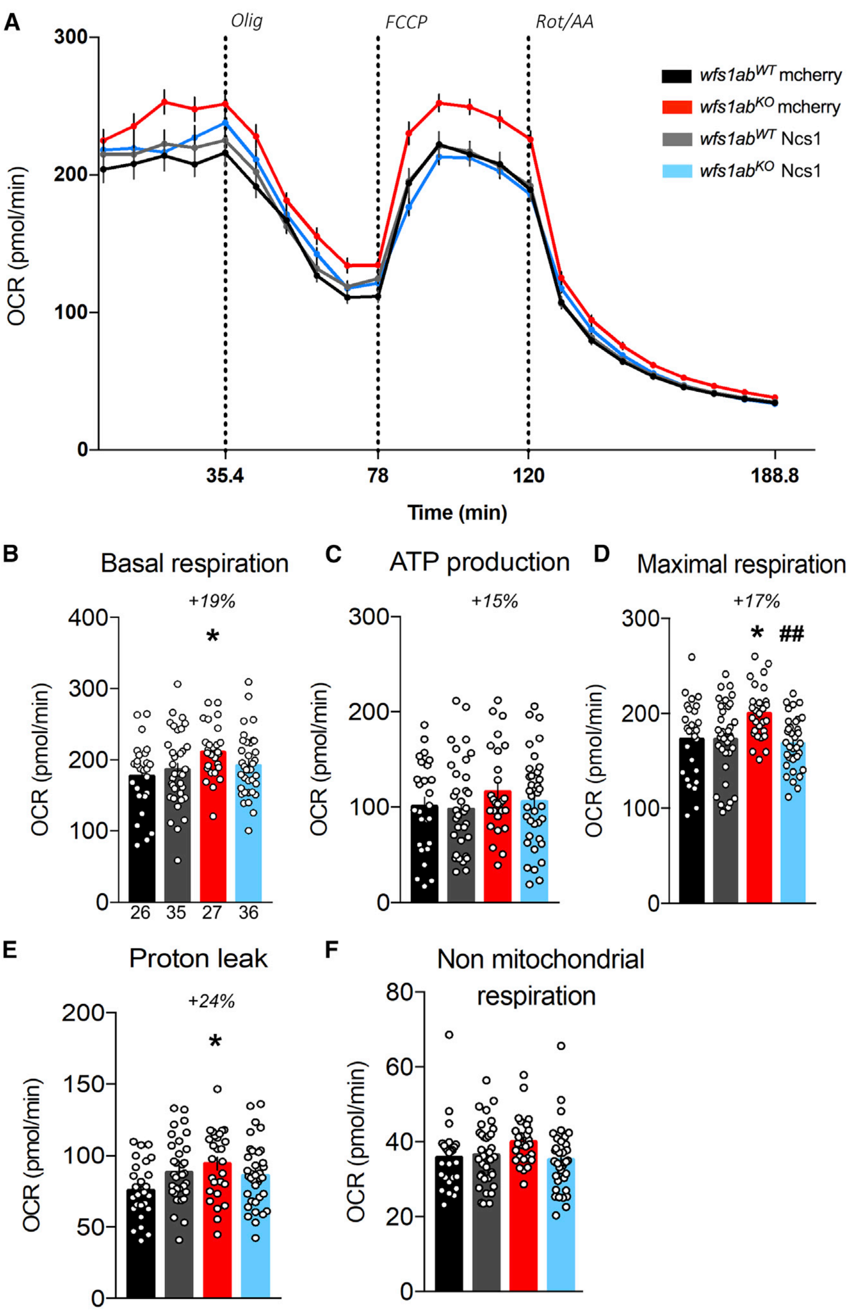

Figure 6. Ncs1 overexpression restores the mitochondrial function

(A) OCR in mCherry- or *Ncs1* RNA-injected $wfs1ab^{WT}$ and $wfs1ab^{KO}$ zebrafish larvae at 5 dpf. (B) Basal respiration, (C) ATP production-related OCR, (D) maximal respiration, (E) proton leak, and (F) non-mitochondrial respiration. Data in (A–F) are mean ± SEM calculated from the number of animals indicated below the columns in (B), n = 26 animals $wfs1ab^{WT}$ mcherry, n = 35 animals $wfs1ab^{KO}$ mcherry, n = 27 animals $wfs1ab^{WT}$ Ncs1, n = 36 animals $wfs1ab^{KO}$ Ncs1. *p < 0.05 versus $wfs1ab^{WT}$, $^{\#\#}$p < 0.01 versus mCherry RNA treatment; Tukey's multiple comparison test.

previously ($wfs1a^{L692X}$,[25] $wfs1a^{C825X}$,[24] and $wfs1b^{W493X}$[24,25]), the complete abolition of Wfs1 functional protein in the $wfs1ab^{KO}$ line was expected to avoid any functional redundancy and compensatory mechanisms and to mimic as closely as possible the human pathology and underlying molecular pathophysiological mechanisms.

Based on our previous study,[24] we intercrossed the $wfs1a^{C825X}$ and $wfs1b^{W493X}$ mutant lines to generate a double-mutant zebrafish line. To assess the role of Wfs1 protein in zebrafish, we initially selected two lines from the ZFIN repository, which were generated through ENU mutagenesis.[26] Both lines carry a point mutation coding for a premature stop codon, potentially leading to non-functional proteins. Moreover, the chosen variants, $wfs1a^{C825X}$ and $wfs1b^{W493X}$, should mimic human mutations E752X or Q819X for Wfs1a and Q486X for Wfs1b.[27,28] WS is a highly heterogeneous disease in its clinical presentation as well as its genetic causes. Even though only one gene is responsible for WS type 1, more than 200 variants have been reported.[27] So far, studies have failed to establish a clear phenotype-genotype correlation, most likely due to the molecular complexity of *WFS1* gene, the inter- and intra-familial divergences of the clinical characteristics, and the rarity of the disease, leading to small size cohorts.[27,29,30] The type and/or location of the variant cannot predict the associated phenotype; however, the variants have been classified according to their gene and protein alterations.[27] Based on this classification, we can hypothesize that $wfs1a^{C825X}$ and $wfs1b^{W493X}$ might lead to a complete degradation of the Wfs1 proteins.

The absence of Wfs1 protein had no effect on the gross morphology of the zebrafish 5-dpf larva, except for an increased size of the ear area compared with $wfs1ab^{WT}$ control. This anomaly was previously observed in the $wfs1a^{C825X}$ larva.[24] We could not, however, exclude some transient developmental deficits in early stages, as suggested by the study of Cairns et al. in single-mutant zebrafish at age up to 80 hpf.[25] As for both single-gene mutant larvae, no difference in auditory function during the noise stimulation was observed using the ASR test, suggesting that the morphological anomaly measured in both lines was not correlated to a functional alteration.[24] In the retina, in contrast to what we previously reported for both mutant lines $wfs1a^{C825X}$ and $wfs1b^{W493X}$,[24] the number of cones and rods decreased in $wfs1ab^{KO}$ larvae at 5 dpf, suggesting that the photoreceptors were altered. Coherently, a visual alteration

was estimated in the VMR test. Cairns et al. reported that in older fish, 4 and 12 months old, both *wfs1a* and *wfs1b* mutant fish exhibited a decrease of the retinal ganglion cell density, associated with thinner retinal ganglion cell layer and in ganglion cell layer.[25] Taken together, these data suggested that the double-mutant line might undergo accelerated visual degeneration process.

Patients with WS develop both neurodevelopmental and neurodegenerative phenotypes. While this study is focused on Wolfram syndrome zebrafish model larvae and the associated neurodevelopmental defects, we can speculate that older fish may present with neurodegeneration. The study carried out by Cairns et al. in other WS zebrafish models highlighted the evolution of the pathophysiology of the disease with the progression of the visual deficit in 4- and 12-month-old fish at both physiological (*wfs1b*^{W493X}) and cellular levels (*wfs1a*^{L692X} and *wfs1b*^{W493X}).[25] In a 15-month-old WS rat model, in addition to axonal degeneration and disorganization of the myelin in the optic nerves leading to an optic atrophy, a decrease of medullary volume of the brain was reported, showing similarities with the brainstem neurodegeneration observed in WS patients.[31] These two independent studies support our hypothesis, and further investigations will be necessary to conclude if our zebrafish model, in addition to neurodevelopmental defects, presents neurodegeneration. However, we need to consider that zebrafish is also a useful model to study regeneration. Indeed, zebrafish has demonstrated good ability to regenerate axons,[32] retina,[33] and pancreas[34] (see also Massoz et al.[35]). In fact, in our model, the disappearance of hyperactivity in 9-dpf larvae (Figure S3) may be explained either by a regenerative process of neurons, or by the fact that the hyperactivity phenotype is due to a developmental delay that was corrected at 9 dpf, as it has been shown in another model by Del Pozo et al.[36] Consequently, the potential regeneration might be a limitation to the study of neurodegeneration.

WFS1 was described as a modulator of UPR in different models.[8,37–40] In the mutant *wfs1a* line, we previously observed significant alterations in the PERK pathway and the mutant *wfs1b* line showed altered IRE1 and PERK pathways under basal conditions and under ER stress conditions induced by tunicamycin treatment.[24] Therefore, we measured the impact of the abrogation of *wfs1* expression in 5-dpf *wfs1ab*^{KO} larvae on the different UPR markers. We observed that, under basal conditions, *wfs1* genes loss of function only impacted the expression level of *eif2s1* and *chop*. Under ER stress conditions, marginal effects were observed on markers of the PERK pathway, but globally it appeared that the mutations have little impact on ER stress. This questioned the precise role of WFS1 in this critical cellular process in the pathology of WS. The data suggested that the WFS1 loss of function consequence is not an altered activation of the UPR but rather an impairment of the mitochondrial function we demonstrated in human patient fibroblasts,[11] and in *Wfs1*$^{\Delta Exon8}$ mutant mice.[41]

WFS1 is indeed also involved in the regulation of the mitochondrial activity. We previously demonstrated that WFS1 deficiency in human fibroblasts was associated with decreased Ca^{2+} uptake by mitochondria, reduced mitochondrial contact with the ER, and decreased mitochondrial respiration.[11] Therefore, we analyzed the impact of the total loss of function of *wfs1* genes on the different parameters of mitochondrial physiology in the living zebrafish at 5 dpf. *wfs1ab*^{KO} mutant larvae showed an increase in basal, ATP-linked, and maximal respirations, but no difference in proton leak and non-mitochondrial respiration compared with controls. The double-mutant line therefore appeared more altered than single-gene mutants that showed no deficit, in *wfs1a*^{C825X} larvae, or transient decreases of basal, ATP-linked, and maximal respirations, in *wfs1b*^{W493X} at 2 dpf but not 5 dpf.[24] This could be explained by direct compensatory mechanisms between *wfs1a* and *wfs1b* genes in single-mutant models. In the double mutant, neither Wfs1a nor Wfs1b are functional and no compensation or altered expression would interfere. Intriguingly, the results in *wfs1ab*^{KO} mutant zebrafish did not mimic those obtained in WS patient fibroblasts. Indeed, in fibroblasts, a decrease in the mitochondrial activity was observed, whereas an increase of the mitochondrial activity is observed in the *wfs1ab*^{KO} zebrafish.[11] One possible explanation is that, *in vitro*, experimental conditions, including glucose concentration or neurotransmitter levels, are controlled, which is not the case *in vivo*. Increased glucose concentration leads to increased OCR in cell.[42] No differences in basal glucose level were observed in *wfs1ab*^{KO} mutant zebrafish; however, additional analysis, such as glucose tolerance test and glucose level at later time points, may help completely rule out this hypothesis. Another hypothesis is that the dopamine concentration is altered in the *wfs1ab*^{KO} mutant, mimicking what was observed in Wfs1-deficient mice following high K^+ challenge.[43] Since it has been shown that an L-DOPA treatment inhibited complex I and ATP levels in the striatum,[44] and dopamine treatment in neuronal cell line induced a reduction in cellular ATP levels,[45] the deficit in neurotransmitter would result *in vivo* in *wfs1ab*^{KO} mutant zebrafish, in an increase in OCR. Further experiments would be needed to check this hypothesis, such as measuring the levels in dopamine and its metabolites in 5-dpf zebrafish larvae, which remains a tricky experiment.

As mentioned above, ER stress plays a key role in WS physiopathology leading to therapeutic development targeting mainly ER stress modulation and regulation. This encompasses chemical chaperone, ER calcium stabilizers, and mitochondrial modulator.[46] However, we and others broaden the spectrum of potential underlying mechanisms, including now calcium dysregulation, as elegantly highlighted by two recent reviews.[47,48] Our choice of NCS1 as a relevant target falls within the scope of this new strategy as we expect NCS1 overexpression to restore the associated Ca^{2+} pathways. In addition, deciphering WFS1-dependent calcium mechanisms may provide additional therapeutic targets. NCS1 is a calcium sensor expressed predominantly in neurons that regulates many cellular functions, including neurotransmission, synaptic plasticity, exocytosis and endocytosis, neuronal growth, neuroprotection, and nuclear Ca^{2+} regulation. Interestingly, NCS1 exerts similar functions as WFS1: both proteins regulate cytosolic Ca^{2+} levels and IP3R-dependent ER-Ca^{2+} release[20,32–34] modulate neuronal morphology and neurodevelopment,[49,50] mediate neuroprotection,[51] and have been implicated

in neurodegenerative diseases and psychiatric disorders beyond WS.[52–54] In human fibroblasts, NCS1 knockdown impaired Ca^{2+} homeostasis and mitochondrial function, demonstrating that NCS1 modulated $[Ca^{2+}]_m$ uptake and could be a target to maintain mitochondrial function and MAM integrity. In addition, the overexpression of NCS1 in patient fibroblasts restored the defective mitochondrial phenotype of WFS1-deficient cells.[11] Moreover, in rat insulinoma cells, the overexpression of NCS1 in *wfs1*^{KO} cells was also shown to restore calcium homeostasis including ATP-evoked ER calcium release and resting cytosolic calcium.[22]

Therefore, we investigated if the overexpression of Ncs1 would bypass Wfs1 activation in our WS zebrafish model. Like the *wfs1* gene, there are two copies of *ncs1* in zebrafish: *ncs1a* and *ncs1b*. We decided to inject the murine *Ncs1* gene, using a sequence that has been optimized for its expression in the zebrafish.[55] Even though *ncs1* is not downregulated in our *wfs1ab*^{KO} mutant zebrafish, murine Ncs1 overexpression, using mRNA microinjection at the embryonic stage, seems to compensate the absence of Wfs1 proteins and counteracts its negative impacts. We cannot exclude, though, that Ncs1-mediated effects would still be through its interaction with Wfs1. Indeed, Ncs1 interacts, *in vivo*, with the N-terminal part (amino acids 1–311) of Wfs1.[11] As stop codons for both lines are located after this domain (C825X and W493X for *wfs1a* and *wfs1b*, respectively), our mutants may still express the N-terminal parts of both *Wfs1* proteins.

Interestingly, the locomotor hyperactivity of 5-dpf *wfs1ab*^{KO} larvae, measured using the VMR in the OFF phase, was reversed after overexpression of Ncs1. More specifically, the abrogation of Wfs1 impacts the mitochondrial activity of 5-dpf *wfs1ab*^{KO} larvae with a significant increase in basal and maximal respiration compared with controls. These alterations were partially or totally reversed by the overexpression of Ncs1. Also, the trends for increased ATP production and non-mitochondrial respiration in *wfs1ab*^{KO} larvae were attenuated by overexpression of Ncs1. We can conclude that the absence of functional Wfs1 led to a mitochondrial alteration and resulted in a locomotor change, i.e., hyperlocomotion. Notably, all these alterations were compensated by the overexpression of Ncs1. Restoration of the locomotor and visual behaviors in mutant larvae appeared directly correlated to the initial restoration of the mitochondrial alteration induced by the overexpression of Ncs1 observed by measuring the OCR.

To date no treatment of WS is available and the on-going clinical trials have not proven efficient yet.[56] Several models for WS have been developed, including IPSC-derived cells,[57] *Drosophila*,[37] zebrafish,[24,25] and rodents (mouse[41,58–60] and rat[31]). None of these models present with all the characteristics of the human pathology and the use of one model over another is dictated by different parameters. Zebrafish is a powerful model to screen drugs *in vivo*. Its inexpensive husbandry and maintenance associated with its small size and high fecundity facilitate its use for large-scale high-throughput drug screening. However, some drawbacks need to be taken in account, such as anatomical and physiological divergences, duplication of the genome that might prevent efficient gene therapy, as well as a limited panel of complex behavioral analysis.[23] This model still remains a model of choice for early-stage high-throughput drug screening, complementary to rodent models. With our double-mutant model, we overcome the limitation due to the duplication of the zebrafish genome, allowing for gene therapy strategy. Moreover, the VMR defect, characteristic of our fish, is a readout that can be easily and extensively used in the context of drug screening.

While the use of NCS1 is currently explored in other indications such as cancer and fragile X syndrome,[61–63] developing therapies with NCS1 as a target in the context of WS is new, and only cell-based studies have been performed so far.[11,22] In the context of rat insulinoma cells, deficient for *Wfs1*, ibudilast, a canonical PDE4 inhibitor and NCS1 binding drug, has been used as an efficient treatment to restore calcium homeostasis, cell viability, and glucose-stimulated insulin secretion.[22] The effective use of a drug targeting NCS1, such as ibudilast, strengthens our strategy to use NCS1 as a therapeutical target. Our study is the first one to use successfully NCS1 in a whole organism. Based on our results and already published studies, we can envision both gene therapy and a pharmaceutical approach with small molecules.

In conclusion, we have characterized a zebrafish model for WS, bearing a double mutation on *wfs1a* and *wfs1b* genes, which will be a pertinent model for drug screening. We have demonstrated *in vivo* the therapeutic potential of NCS1 overexpression as a first stone to pave the way to an efficient therapeutic strategy that could halt the progression of WS disease.

MATERIALS AND METHODS

Zebrafish lines and husbandry

This study followed the recommendations of the ARRRIVE guidelines[64] and the European Union Directive 2010/63. The *wfs1ab*^{KO} mutant line was generated by cross-breeding the two lines *wfs1a*^{C825X} and *wfs1b*^{W493X}, as described in Crouzier et al.[24] Adult zebrafish were bred and maintained under standard conditions in an automated fish tank system (ZebTEC, Tecniplast, Louviers, France) at 28°C, pH 7, conductivity around 500 mS, and with a 14:10 h light:dark cycle. Eggs were obtained by natural spawning and maintained in fish water at 28°C. Each experimental procedure was carried out in triplicate and larvae were from three different crosses.

Chemical treatment

To induce ER stress, larvae were incubated, at 4 dpf, for 24 h with 2 μg/mL of tunicamycin (sc-3606, Santa Cruz Biotechnology, Dallas, TX), and diluted in 0.1% dimethylsulfoxide (DMSO) directly in the fish water. Control larvae were treated with 0.1% DMSO diluted in fish water.

mRNA injection

Transcripts encoding mouse wild-type Ncs1 were generated after insertion of the murine *Ncs1* coding sequence in the pcDNA3.1(+)

plasmid (construct engineered by GenScript, Leiden, the Netherlands). The sequence was optimized for a better translation in the zebrafish (sequence available upon request). The *Ncs1* mRNAs were transcribed from Not1-linearized pcDNA3.1(+) using the T7 polymerase with the mMESSAGE Machine kit (Ambion, Austin, TX) and purified accordingly to the manufacturer's instructions. Eight hundred nanograms ng of *Ncs1* RNAs, in a 1 nL volume, was microinjected into one-cell- to two-cell-stage embryos according to standard protocols. mCherry RNAs from pCS2+-mCherry were used as an injection control (generous gift from Dr G. Lutfalla, LPHI, Montpellier, France).

RT-PCR and quantitative real-time PCR

At 5 dpf, total RNA from 20 whole $wfs1ab^{KO}$ mutant larvae and their associated controls were extracted using a Nucleospin RNA Kit (Macherey-Nagel, Hoerdt, France) according to the manufacturer's instructions. RNA concentration and purity were evaluated using the Agilent RNA 6000 Nano Kit (Agilent Technologies, Santa Clara, CA). RNA samples (1 μg) were denatured for 5 min at 70°C and reverse transcribed into cDNA for 1 h at 37°C, using M-MLV reverse transcriptase (Promega, Madison, WI). Primer sequences are detailed in the supplemental information in Table S1. Control reactions were conducted with sterile water to determine signal background and DNA contamination. The standard curve of each gene was confirmed to be in a linear range, while *zef1α* gene was selected as reference.

VMR assay

The locomotor activity of zebrafish larvae was quantified by VMR using an infrared (IR) tracking system (Zebralab, Viewpoint, Lissieux, France) as described previously.[65] In brief, at 5 dpf, larvae were transferred into a 96-well plate (Whatman, no. 7701-1651) with 300 μL fish water and the locomotor behavior was monitored with an automated videotracking device (Zebrabox, ViewPoint). The response to light changes was recorded by an IR camera under IR light illumination. The light protocol was as follows: 30 min of acclimatization in the dark (0% light intensity), then two cycles of 10-min duration light ON (100% light intensity) or light OFF (0%) periods. The total duration of the experiment was 70 min. Activity during the experiment was measured in mm/s. The values obtained during OFF periods were subtracted for each larva from their values registered during ON periods to remove inter- and intragroup variability in basic locomotion.

ASR assay

The locomotor activity of zebrafish larvae was quantified using ASR in the ZebraBox. Experimental conditions were similar to those used in the VMR assay. The experiment consisted, first, in acclimating larvae during 30 min with no sound (35 dB ambient), followed by a 1-s stimulation with white sound at 90 dB, repeated three times with an intertrial time interval of 5 min. The quantity of movements during the entire experiment was measured for each larva. Baseline activity levels were subtracted from the activity levels during the sound stimulations (2 min before each stimulation) to normalize the values.

OKR assay

At 5-dpf zebrafish larvae were immersed per group of four in a Petri dish (35 mm diameter) containing 2.5% methylcellulose (no. 9004-65-3, Sigma Aldrich, St. Louis, MI). Larvae were placed dorsal up and forming an X to avoid touching and interfering with each other. All measurements were done in the afternoon between 2:00 p.m. and 6:00 p.m. The room temperature was 28°C and the light was OFF. Visual system performance of larval zebrafish was assessed using a videotracking device (VisioBox, ViewPoint). Forty 6-mm-wide black and white strips were projected at 2 rpm for 1 min clockwise and then 1 min anti-clockwise. The IR illuminated larvae from the bottom and responses were tracked using an FL3-U3-32S2M 1/2.8-inch Monochrome camera (FLEA3, Flir) at 25 frames/s. The number of saccades was manually recorded (PHIVisualize software) and the average number of saccades per 2 min quantified.

Touch-escape response

To measure the touch-escape, 5-dpf larvae were transferred to a rail developed with a 3D printer (18 × 0.4 cm) with 200 μL of fish water and placed at the extremity of the rail. The tail of larvae was touched with a tip and the distance traveled was measured during 5 s. The same procedure was repeated three times per larvae, each test separated by 1 min to reduce stress. Each larva was assessed individually and the three values per larva were averaged. The light was ON and the temperature was 28°C.

Immunohistochemistry

Whole larvae were fixed in paraformaldehyde at 4°C for 48 h, cryoprotected in 30% sucrose and mounted in O.C.T. medium (Sakura, Tissue-Tek, Alphen aan den Rijn, the Netherlands). Larvae were transversely sectioned in 10-μm-thick slices using a cryostat (Leica, Wetzlar, Germany) at −20°C and mounted on glass slides. Cryosections were blocked with a solution containing 0.1% phosphate buffered saline/Triton X-100 and 5% horse serum for 30 min at room temperature. They were subsequently incubated at 4°C overnight with the following primary antibodies: mouse anti-Rho4d2 (1:7,000; ab98887, Abcam, Cambridge, UK) and mouse anti-Zpr-1 (1:500; ab174435, Abcam). After several washes, sections were incubated with specific secondary antibodies: Cy3 conjugated anti-mouse antibody (1:800; 715-165-150, Jackson ImmunoResearch, West Grove, PA), Cy3-conjugated anti-rabbit antibody (1:1,000; 711-166-152, Jackson ImmunoResearch), or Alexa Fluor 488 conjugated anti-mouse antibody (1:1,000; 715-545-150, Jackson ImmunoResearch). Nuclei were counterstained with 40,6-diamidino-2 phenylendole (DAPI) (1:5,000; Sigma Aldrich). The emitted fluorescence was measured using a confocal microscope (LSM880 Fastairyscan, Carl Zeiss, Jena, Germany).

Cell counts

Cone cells, immunolabeled with Zpr-1 antibody, were quantified individually and the total area of rod outer segments, immunolabeled with Rho4d2 antibody, was evaluated and both measures were normalized to the length of the associated retina. Ganglion cells, highlighted by DAPI counterstaining, were counted in three specific

regions of the retina, consistent from one sample to another. The total number of ganglion cells from all regions was averaged per larva. The thickness of the ganglion cell layer was also quantified in the same specific regions of the retina.

Measurement of total free glucose levels

At 5 dpf, 10 larvae were homogenized in 5 μL of NaCl 0.9% using a hand homogenizer, and centrifuged at 21,694 × *g* for 2 min. The supernatant (1.5 μL) was placed on a glucometer strip (Accu-Chek Performa). Total free glucose levels were measured using a glucometer (Accu-Chek Performa Nano) and two measurements were averaged per sample.

Seahorse XFe24 MitoStress test

The OCR of 5-dpf larvae was measured with the Seahorse XFe24 extracellular flux analyzer (Agilent, Santa Clara, CA). Larvae were singly placed in wells of a Seahorse XFe24 spheroid microplate, containing 500 μL of fish water. A grid was placed manually on larvae to keep them at the bottom of the wells throughout the experiment. The blanks were two empty wells of the plate. Four basal cycles readings were recorded, then five recording cycles following oligomycin (25 μM) injection, five recordings cycles after carbonyl cyanide-p-trifluoromethoxyphenylhydrazone (FCCP) (8 μM) injection and nine recordings cycles after rotenone + antimycin A (1.5 μM) injection. Calculations for specific parameters (non-mitochondrial respiration, basal respiration, maximal respiration, proton leak, and ATP production) were made. The room temperature was controlled at 28°C. Measurements of total zebrafish OCR were started immediately and performed according to the manufacturer's instructions.

Western blot

To measure protein expression, 20 whole larvae per condition, at 5 dpf, were homogenized on ice for 15 s in 100 μL of lysis buffer (62.5 mM Tris-HCl, 25% glycerol, 2% sodium dodecyl sulfate, 0.04% cOmplete, 0.1% PhosSTOP [pH 6.8]). Total proteins were separated on a 1.5-mm 12% acrylamide running gel and 4% acrylamide stacking gel at 100 V. Proteins were transferred into a nitrocellulose membrane at 100 V for 1 h in transfer buffer and blocked with 5% non-fat milk solution for 1 h. Immunoblotting was performed with primary antibodies as follows: rabbit anti-Sigmar1 antibody (1:500, 15168-1-AP; Proteintech), rabbit anti-GADD153 antibody (1:1,000, G6916, Sigma Aldrich), rabbit anti-Bip antibody (1:700, SPC-180, Biosciences), rabbit anti-Eif2α antibody (1:500, no. 9722, Cell Signaling), and rabbit anti-p-Eif2α antibody (1:500, no. 3398, Cell Signaling), in buffer (0.1% TBS/Triton X-100) pH 7.4, overnight at 4°C. After several washes, membranes were incubated with horseradish peroxidase (HRP)-conjugated goat anti-rabbit secondary antibody (1:2,000; ab6721, Abcam) or goat anti-mouse (1:2,000; ab6789, Abcam) secondary antibody for 1 h at room temperature and the proteins were detected with the indicated HRP detection reagent (10776189, Merck) and the Bio-Rad imaging system. Relative intensities of each band were quantified using Image Lab v.6.1 software (Bio-Rad, Hercules, CA) and normalized to the total protein quantity (Stain-Free, Bio-Rad) (Figure S4).

Statistical analyses

Data are expressed as mean ± SEM. Statistical significance between groups was determined by unpaired Student's t test or two-way ANOVA. The levels of statistical significance considered were: $^{*}p < 0.05$, $^{**}p < 0.01$, and $^{***}p < 0.001$. Statistical analyses were performed using the Prism v.7.0 software (GraphPad, San Diego, CA). Two-way ANOVA statistical values for Figure 5 (Table S2) and for Figure 6 (Table S3) are presented in the supplemental information.

DATA AND MATERIALS AVAILABILITY

All of the data and materials are available upon request.

SUPPLEMENTAL INFORMATION

Supplemental information can be found online at https://doi.org/10.1016/j.omtm.2022.10.003.

ACKNOWLEDGMENTS

We acknowledge the imaging facility MRI, member of the national infrastructure France-BioImaging supported by the French National Research Agency (ANR-10-INBS-04, "Investments for the future"). We thank the *ZebraSens* zebrafish phenotyping platform at MMDN (Montpellier, France) and the qPHd quantitative PCR platform at University of Montpellier (Montpellier, France). This work was supported in part by external resources of the University of Montpellier (Fondation Pour l'Audition FPA RD-2019-13, Retina France Association and Foundation). L.C. was supported by Association syndrome de Wolfram and the Région Occitanie and E.M.R. by The Snow Foundation and Eye Hope Foundation.

AUTHOR CONTRIBUTIONS

B.D. and T.M. conceived the project. B.D. and T.M. designed the experiments and analyzed the data. L.C., E.M.R., C.D., M.D., A.P., and H.A. performed the experiments and analyses. N.C. took care of the zebrafish. T.D. helped with novel ASR device. L.C., E.M.R., B.D., and T.M. prepared the figures, and wrote and edited the manuscript. All authors corrected and approved the manuscript and the figures.

DECLARATION OF INTERESTS

B.D. holds a patent "Targeting the neuronal calcium sensor 1 for treating Wolfram syndrome." 2016-03-23 EP16305330.9, WO2017162798A1. The other authors declare no competing interests.

REFERENCES

1. Barrett, T.G., Bundey, S.E., and Macleod, A.F. (1995). Neurodegeneration and diabetes: UK nationwide study of Wolfram (DIDMOAD) syndrome. Lancet Lond. Engl. *346*, 1458–1463. https://doi.org/10.1016/s0140-6736(95)92473-6.

2. Kinsley, B.T., Swift, M., Dumont, R.H., and Swift, R.G. (1995). Morbidity and mortality in the wolfram syndrome. Diabetes Care *18*, 1566–1570. https://doi.org/10.2337/diacare.18.12.1566.

3. Mozzillo, E., Delvecchio, M., Carella, M., Grandone, E., Palumbo, P., Salina, A., Aloi, C., Buono, P., Izzo, A., D'Annunzio, G., et al. (2014). A novel CISD2 intragenic deletion, optic neuropathy and platelet aggregation defect in Wolfram syndrome type 2. BMC Med. Genet. *15*, 88. https://doi.org/10.1186/1471-2350-15-88.

4. Amr, S., Heisey, C., Zhang, M., Xia, X.-J., Shows, K.H., Ajlouni, K., Pandya, A., Satin, L.S., El-Shanti, H., and Shiang, R. (2007). A homozygous mutation in a novel

zinc-finger protein, ERIS, is responsible for Wolfram syndrome 2. Am. J. Hum. Genet. *81*, 673–683. https://doi.org/10.1086/520961.

5. Ajlouni, K., Jarrah, N., El-Khateeb, M., El-Zaheri, M., El Shanti, H., and Lidral, A. (2002). Wolfram syndrome: identification of a phenotypic and genotypic variant from Jordan. Am. J. Med. Genet. *115*, 61–65. https://doi.org/10.1002/ajmg.10345.

6. Inoue, H., Tanizawa, Y., Wasson, J., Behn, P., Kalidas, K., Bernal-Mizrachi, E., Mueckler, M., Marshall, H., Donis-Keller, H., Crock, P., et al. (1998). A gene encoding a transmembrane protein is mutated in patients with diabetes mellitus and optic atrophy (Wolfram syndrome). Nat. Genet. *20*, 143–148. https://doi.org/10.1038/2441.

7. Strom, T.M., Hörtnagel, K., Hofmann, S., Gekeler, F., Scharfe, C., Rabl, W., Gerbitz, K.D., and Meitinger, T. (1998). Diabetes insipidus, diabetes mellitus, optic atrophy and deafness (DIDMOAD) caused by mutations in a novel gene (wolframin) coding for a predicted transmembrane protein. Hum. Mol. Genet. *7*, 2021–2028. https://doi.org/10.1093/hmg/7.13.2021.

8. Fonseca, S.G., Ishigaki, S., Oslowski, C.M., Lu, S., Lipson, K.L., Ghosh, R., Hayashi, E., Ishihara, H., Oka, Y., Permutt, M.A., et al. (2010). Wolfram syndrome 1 gene negatively regulates ER stress signaling in rodent and human cells. J. Clin. Invest. *120*, 744–755. https://doi.org/10.1172/JCI39678.

9. Pallotta, M.T., Tascini, G., Crispoldi, R., Orabona, C., Mondanelli, G., Grohmann, U., and Esposito, S. (2019). Wolfram syndrome, a rare neurodegenerative disease: from pathogenesis to future treatment perspectives. J. Transl. Med. *17*, 238. https://doi.org/10.1186/s12967-019-1993-1.

10. Delprat, B., Maurice, T., and Delettre, C. (2018). Wolfram syndrome: MAMs' connection? Cell Death Dis. *9*, 364. https://doi.org/10.1038/s41419-018-0406-3.

11. Angebault, C., Fauconnier, J., Patergnani, S., Rieusset, J., Danese, A., Affortit, C.A., Jagodzinska, J., Mégy, C., Quiles, M., Cazevieille, C., et al. (2018). ER-mitochondria cross-talk is regulated by the Ca2+ sensor NCS1 and is impaired in Wolfram syndrome. Sci. Signal. *11*. https://doi.org/10.1126/scisignal.aaq1380.

12. Bourne, Y., Dannenberg, J., Pollmann, V., Marchot, P., and Pongs, O. (2001). Immunocytochemical localization and crystal structure of human Frequenin (neuronal calcium sensor 1). J. Biol. Chem. *276*, 11949–11955. https://doi.org/10.1074/jbc.M009373200.

13. Burgoyne, R.D., Helassa, N., McCue, H.V., and Haynes, L.P. (2019). Calcium sensors in neuronal function and dysfunction. Cold Spring Harb. Perspect. Biol. *11*, a035154. https://doi.org/10.1101/cshperspect.a035154.

14. McFerran, B.W., Graham, M.E., and Burgoyne, R.D. (1998). Neuronal Ca2+ sensor 1, the mammalian homologue of Frequenin, is expressed in chromaffin and PC12 cells and regulates neurosecretion from dense-core granules. J. Biol. Chem. *273*, 22768–22772. https://doi.org/10.1074/jbc.273.35.22768.

15. Dason, J.S., Romero-Pozuelo, J., Marin, L., Iyengar, B.G., Klose, M.K., Ferrús, A., and Atwood, H.L. (2009). Frequenin/NCS-1 and the Ca2+-channel α1-subunit co-regulate synaptic transmission and nerve-terminal growth. J. Cell Sci. *122*, 4109–4121. https://doi.org/10.1242/jcs.055095.

16. Romero-Pozuelo, J., Dason, J.S., Atwood, H.L., and Ferrús, A. (2007). Chronic and acute alterations in the functional levels of Frequenins 1 and 2 reveal their roles in synaptic transmission and axon terminal morphology. Eur. J. Neurosci. *26*, 2428–2443. https://doi.org/10.1111/j.1460-9568.2007.05877.x.

17. Hui, K., Fei, G.-H., Saab, B.J., Su, J., Roder, J.C., and Feng, Z.-P. (2007). Neuronal calcium sensor-1 modulation of optimal calcium level for neurite outgrowth. Dev. Camb. Engl. *134*, 4479–4489. https://doi.org/10.1242/dev.008979.

18. Gomez, M., De Castro, E., Guarin, E., Sasakura, H., Kuhara, A., Mori, I., Bartfai, T., Bargmann, C.I., and Nef, P. (2001). Ca2+ signaling via the neuronal calcium sensor-1 regulates associative learning and memory in C. elegans. Neuron *30*, 241–248. https://doi.org/10.1016/S0896-6273(01)00276-8.

19. Saab, B.J., Georgiou, J., Nath, A., Lee, F.J.S., Wang, M., Michalon, A., Liu, F., Mansuy, I.M., and Roder, J.C. (2009). NCS-1 in the dentate gyrus promotes exploration, synaptic plasticity, and rapid acquisition of spatial memory. Neuron *63*, 643–656. https://doi.org/10.1016/j.neuron.2009.08.014.

20. Reynolds, A.J., Bartlett, S.E., and Morgans, C. (2001). The distribution of neuronal calcium sensor-1 protein in the developing and adult rat retina. Neuroreport *12*, 725–728. https://doi.org/10.1097/00001756-200103260-00022.

21. Gromada, J., Bark, C., Smidt, K., Efanov, A.M., Janson, J., Mandic, S.A., Webb, D.-L., Zhang, W., Meister, B., Jeromin, A., et al. (2005). Neuronal calcium sensor-1 potentiates glucose-dependent exocytosis in pancreatic beta cells through activation of phosphatidylinositol 4-kinase beta. Proc. Natl. Acad. Sci. USA *102*, 10303–10308. https://doi.org/10.1073/pnas.0504487102.

22. Nguyen, L.D., Fischer, T.T., Abreu, D., Arroyo, A., Urano, F., and Ehrlich, B.E. (2020). Calpain inhibitor and ibudilast rescue β cell functions in a cellular model of Wolfram syndrome. Proc. Natl. Acad. Sci. USA *117*, 17389–17398. https://doi.org/10.1073/pnas.2007136117.

23. Crouzier, L., Richard, E.M., Sourbron, J., Lagae, L., Maurice, T., and Delprat, B. (2021). Use of zebrafish models to boost research in rare genetic diseases. Int. J. Mol. Sci. *22*, 13356. https://doi.org/10.3390/ijms222413356.

24. Crouzier, L., Richard, E.M., Diez, C., Alzaeem, H., Denus, M., Cubedo, N., Delaunay, T., Glendenning, E., Baxendale, S., Liévens, J.-C., et al. (2022). Morphological, behavioral and cellular analyses revealed different phenotypes in Wolfram syndrome wfs1a and wfs1b zebrafish mutant lines. Hum. Mol. Genet. ddac065. https://doi.org/10.1093/hmg/ddac065.

25. Cairns, G., Burté, F., Price, R., O'Connor, E., Toms, M., Mishra, R., Moosajee, M., Pyle, A., Sayer, J.A., and Yu-Wai-Man, P. (2021). A mutant wfs1 zebrafish model of Wolfram syndrome manifesting visual dysfunction and developmental delay. Sci. Rep. *11*, 20491. https://doi.org/10.1038/s41598-021-99781-0.

26. Kettleborough, R.N.W., Busch-Nentwich, E.M., Harvey, S.A., Dooley, C.M., de Bruijn, E., van Eeden, F., Sealy, I., White, R.J., Herd, C., Nijman, I.J., et al. (2013). A systematic genome-wide analysis of zebrafish protein-coding gene function. Nature *496*, 494–497. https://doi.org/10.1038/nature11992.

27. Rigoli, L., Bramanti, P., Di Bella, C., and De Luca, F. (2018). Genetic and clinical aspects of Wolfram syndrome 1, a severe neurodegenerative disease. Pediatr. Res. *83*, 921–929. https://doi.org/10.1038/pr.2018.17.

28. De Franco, E., Flanagan, S.E., Yagi, T., Abreu, D., Mahadevan, J., Johnson, M.B., Jones, G., Acosta, F., Mulaudzi, M., Lek, N., et al. (2017). Dominant ER stress-inducing WFS1 mutations underlie a genetic syndrome of neonatal/infancy-onset diabetes, congenital sensorineural deafness, and congenital cataracts. Diabetes *66*, 2044–2053. https://doi.org/10.2337/db16-1296.

29. de Heredia, M.L., Clèries, R., and Nunes, V. (2013). Genotypic classification of patients with Wolfram syndrome: insights into the natural history of the disease and correlation with phenotype. Genet. Med. Off. J. Am. Coll. Med. Genet. *15*, 497–506. https://doi.org/10.1038/gim.2012.180.

30. Delvecchio, M., Iacoviello, M., Pantaleo, A., and Resta, N. (2021). Clinical spectrum associated with Wolfram syndrome type 1 and type 2: a review on genotype-phenotype correlations. Int. J. Environ. Res. Public. Health *18*, 4796. https://doi.org/10.3390/ijerph18094796.

31. Plaas, M., Seppa, K., Reimets, R., Jagomäe, T., Toots, M., Koppel, T., Vallisoo, T., Nigul, M., Heinla, I., Meier, R., et al. (2017). Wfs1- deficient rats develop primary symptoms of Wolfram syndrome: insulin-dependent diabetes, optic nerve atrophy and medullary degeneration. Sci. Rep. *7*, 10220. https://doi.org/10.1038/s41598-017-09392-x.

32. Tsata, V., and Wehner, D. (2021). Know how to regrow-axon regeneration in the zebrafish spinal cord. Cells *10*, 1404. https://doi.org/10.3390/cells10061404.

33. Stella, S.L., Geathers, J.S., Weber, S.R., Grillo, M.A., Barber, A.J., Sundstrom, J.M., and Grillo, S.L. (2021). Neurodegeneration, neuroprotection and regeneration in the zebrafish retina. Cells *10*, 633. https://doi.org/10.3390/cells10030633.

34. Matsuda, H. (2018). Zebrafish as a model for studying functional pancreatic β cells development and regeneration. Dev. Growth Differ. *60*, 393–399. https://doi.org/10.1111/dgd.12565.

35. Massoz, L., Dupont, M.A., and Manfroid, I. (2021). Zebra-fishing for regenerative awakening in mammals. Biomedicines *9*, 65. https://doi.org/10.3390/biomedicines9010065.

36. Del Pozo, A., Manuel, R., Iglesias Gonzalez, A.B., Koning, H.K., Habicher, J., Zhang, H., Allalou, A., Kullander, K., and Boije, H. (2020). Behavioral characterization of dmrt3a mutant zebrafish reveals crucial aspects of vertebrate locomotion through phenotypes related to acceleration. eNeuro *7*. ENEURO.0047-20. https://doi.org/10.1523/ENEURO.0047-20.2020.

37. Sakakibara, Y., Sekiya, M., Fujisaki, N., Quan, X., and Iijima, K.M. (2018). Knockdown of wfs1, a fly homolog of Wolfram syndrome 1, in the nervous system increases susceptibility to age- and stress-induced neuronal dysfunction and

degeneration in Drosophila. PLoS Genet. *14*, e1007196. https://doi.org/10.1371/journal.pgen.1007196.

38. Shang, L., Hua, H., Foo, K., Martinez, H., Watanabe, K., Zimmer, M., Kahler, D.J., Freeby, M., Chung, W., LeDuc, C., et al. (2014). β-cell dysfunction due to increased ER stress in a stem cell model of Wolfram syndrome. Diabetes *63*, 923–933. https://doi.org/10.2337/db13-0717.

39. Guo, X., Shen, S., Song, S., He, S., Cui, Y., Xing, G., Wang, J., Yin, Y., Fan, L., He, F., et al. (2011). The E3 ligase Smurf1 regulates Wolfram syndrome protein stability at the endoplasmic reticulum. J. Biol. Chem. *286*, 18037–18047. https://doi.org/10.1074/jbc.M111.225615.

40. Fonseca, S.G., Fukuma, M., Lipson, K.L., Nguyen, L.X., Allen, J.R., Oka, Y., and Urano, F. (2005). WFS1 is a novel component of the unfolded protein response and maintains homeostasis of the endoplasmic reticulum in pancreatic beta-cells. J. Biol. Chem. *280*, 39609–39615. https://doi.org/10.1074/jbc.M507426200.

41. Crouzier, L., Danese, A., Yasui, Y., Richard, E.M., Liévens, J.-C., Patergnani, S., Couly, S., Diez, C., Denus, M., Cubedo, N., et al. (2022). Activation of the sigma-1 receptor chaperone alleviates symptoms of Wolfram syndrome in preclinical models. Sci. Transl. Med. *14*, eabh3763. https://doi.org/10.1126/scitranslmed.abh3763.

42. Wang, W., Upshaw, L., Strong, D.M., Robertson, R.P., and Reems, J. (2005). Increased oxygen consumption rates in response to high glucose detected by a novel oxygen biosensor system in non-human primate and human islets. J. Endocrinol. *185*, 445–455. https://doi.org/10.1677/joe.1.06092.

43. Matto, V., Terasmaa, A., Vasar, E., and Kõks, S. (2011). Impaired striatal dopamine output of homozygous Wfs1 mutant mice in response to [K+] challenge. J. Physiol. Biochem. *67*, 53–60. https://doi.org/10.1007/s13105-010-0048-0.

44. Przedborski, S., Jackson-Lewis, V., Muthane, U., Jiang, H., Ferreira, M., Naini, A.B., and Fahn, S. (1993). Chronic levodopa administration alters cerebral mitochondrial respiratory chain activity. Ann. Neurol. *34*, 715–723. https://doi.org/10.1002/ana.410340515.

45. Ben-Shachar, D., Zuk, R., Gazawi, H., and Ljubuncic, P. (2004). Dopamine toxicity involves mitochondrial complex I inhibition: implications to dopamine-related neuropsychiatric disorders. Biochem. Pharmacol. *67*, 1965–1974. https://doi.org/10.1016/j.bcp.2004.02.015.

46. Abreu, D., and Urano, F. (2019). Current landscape of treatments for Wolfram syndrome. Trends Pharmacol. Sci. *40*, 711–714. https://doi.org/10.1016/j.tips.2019.07.011.

47. Fischer, T.T., and Ehrlich, B.E. (2020). Wolfram syndrome: a monogenic model to study diabetes mellitus and neurodegeneration. Curr. Opin. Physiol. *17*, 115–123. https://doi.org/10.1016/j.cophys.2020.07.009.

48. Loncke, J., Vervliet, T., Parys, J.B., Kaasik, A., and Bultynck, G. (2021). Uniting the divergent Wolfram syndrome-linked proteins WFS1 and CISD2 as modulators of Ca2+ signaling. Sci. Signal. *14*, eabc6165. https://doi.org/10.1126/scisignal.abc6165.

49. Cagalinec, M., Liiv, M., Hodurova, Z., Hickey, M.A., Vaarmann, A., Mandel, M., Zeb, A., Choubey, V., Kuum, M., Safiulina, D., et al. (2016). Role of mitochondrial dynamics in neuronal development: mechanism for Wolfram syndrome. PLoS Biol. *14*, e1002511. https://doi.org/10.1371/journal.pbio.1002511.

50. Fischer, T.T., Nguyen, L.D., and Ehrlich, B.E. (2021). Neuronal calcium sensor 1 (NCS1) dependent modulation of neuronal morphology and development. FASEB J. *35*. https://doi.org/10.1096/fj.202100731R.

51. Nakamura, T.Y., Jeromin, A., Smith, G., Kurushima, H., Koga, H., Nakabeppu, Y., Wakabayashi, S., and Nabekura, J. (2006). Novel role of neuronal Ca2+ sensor-1 as a survival factor up-regulated in injured neurons. J. Cell Biol. *172*, 1081–1091. https://doi.org/10.1083/jcb.200508156.

52. Cryns, K., Thys, S., Van Laer, L., Oka, Y., Pfister, M., Van Nassauw, L., Smith, R.J.H., Timmermans, J.-P., and Van Camp, G. (2003). The WFS1 gene, responsible for low frequency sensorineural hearing loss and Wolfram syndrome, is expressed in a variety of inner ear cells. Histochem. Cell Biol. *119*, 247–256. https://doi.org/10.1007/s00418-003-0495-6.

53. Bandura, J., and Feng, Z.-P. (2019). Current understanding of the role of neuronal calcium sensor 1 in neurological disorders. Mol. Neurobiol. *56*, 6080–6094. https://doi.org/10.1007/s12035-019-1497-2.

54. Chen, S., Acosta, D., Li, L., Liang, J., Chang, Y., Wang, C., Fitzgerald, J., Morrison, C., Goulbourne, C.N., Nakano, Y., et al. (2022). Wolframin is a novel regulator of tau pathology and neurodegeneration. Acta Neuropathol. *143*, 547–569. https://doi.org/10.1007/s00401-022-02417-4.

55. Horstick, E.J., Jordan, D.C., Bergeron, S.A., Tabor, K.M., Serpe, M., Feldman, B., and Burgess, H.A. (2015). Increased functional protein expression using nucleotide sequence features enriched in highly expressed genes in zebrafish. Nucleic Acids Res. *43*, e48. https://doi.org/10.1093/nar/gkv035.

56. Abreu, D., Stone, S.I., Pearson, T.S., Bucelli, R.C., Simpson, A.N., Hurst, S., Brown, C.M., Kries, K., Onwumere, C., Gu, H., et al. (2021). A phase Ib/IIa clinical trial of dantrolene sodium in patients with Wolfram syndrome. JCI Insight *6*, 145188. https://doi.org/10.1172/jci.insight.145188.

57. Grzela, D.P., Marciniak, B., and Pulaski, L. (2020). Characterization of an induced pluripotent stem cell line (IMBPASi001-A) derived from fibroblasts of a patient affected by Wolfram Syndrome. Stem Cell Res. *46*, 101858. https://doi.org/10.1016/j.scr.2020.101858.

58. Luuk, H., Plaas, M., Raud, S., Innos, J., Sütt, S., Lasner, H., Abramov, U., Kurrikoff, K., Kõks, S., and Vasar, E. (2009). Wfs1-deficient mice display impaired behavioural adaptation in stressful environment. Behav. Brain Res. *198*, 334–345. https://doi.org/10.1016/j.bbr.2008.11.007.

59. Ishihara, H., Takeda, S., Tamura, A., Takahashi, R., Yamaguchi, S., Takei, D., Yamada, T., Inoue, H., Soga, H., Katagiri, H., et al. (2004). Disruption of the WFS1 gene in mice causes progressive beta-cell loss and impaired stimulus-secretion coupling in insulin secretion. Hum. Mol. Genet. *13*, 1159–1170. https://doi.org/10.1093/hmg/ddh125.

60. Bonnet Wersinger, D., Benkafadar, N., Jagodzinska, J., Hamel, C., Tanizawa, Y., Lenaers, G., and Delettre, C. (2014). Impairment of visual function and retinal ER stress activation in Wfs1-deficient mice. PLoS One *9*, e97222. https://doi.org/10.1371/journal.pone.0097222.

61. Benbow, J.H., Mann, T., Keeler, C., Fan, C., Hodsdon, M.E., Lolis, E., DeGray, B., and Ehrlich, B.E. (2012). Inhibition of paclitaxel-induced decreases in calcium signaling. J. Biol. Chem. *287*, 37907–37916. https://doi.org/10.1074/jbc.M112.385070.

62. Bong, A.H.L., Robitaille, M., Milevskiy, M.J.G., Roberts-Thomson, S.J., and Monteith, G.R. (2020). NCS-1 expression is higher in basal breast cancers and regulates calcium influx and cytotoxic responses to doxorubicin. Mol. Oncol. *14*, 87–104. https://doi.org/10.1002/1878-0261.12589.

63. Mansilla, A., Chaves-Sanjuan, A., Campillo, N.E., Semelidou, O., Martínez-González, L., Infantes, L., González-Rubio, J.M., Gil, C., Conde, S., Skoulakis, E.M.C., et al. (2017). Interference of the complex between NCS-1 and Ric8a with phenothiazines regulates synaptic function and is an approach for fragile X syndrome. Proc. Natl. Acad. Sci. USA *114*, E999–E1008. https://doi.org/10.1073/pnas.1611089114.

64. Kilkenny, C., Browne, W.J., Cuthill, I.C., Emerson, M., and Altman, D.G. (2010). Improving bioscience research reporting: the ARRIVE guidelines for reporting animal research. J. Pharmacol. Pharmacother. *1*, 94–99. https://doi.org/10.4103/0976-500X.72351.

65. Crouzier, L., Diez, C., Richard, E.M., Cubedo, N., Barbereau, C., Rossel, M., Delaunay, T., Maurice, T., and Delprat, B. (2021). Loss of Pde6a induces rod outer segment shrinkage and visual alterations in pde6aQ70X mutant zebrafish, a relevant model of retinal dystrophy. Front. Cell Dev. Biol. *9*, 675517. https://doi.org/10.3389/fcell.2021.675517.

OMTM, Volume *27*

Supplemental information

NCS1 overexpression restored mitochondrial activity and behavioral alterations in a zebrafish model of Wolfram syndrome

Lucie Crouzier, Elodie M. Richard, Camille Diez, Morgane Denus, Amandine Peyrel, Hala Alzaeem, Nicolas Cubedo, Thomas Delaunay, Tangui Maurice, and Benjamin Delprat

Table S1. Two-way ANOVA statistical values for Figure 3D.

gene	*Factor*	*F (DFn, DFd)*	*P value*
sigmar1	Genotype	F (1, 16) = 0,2601	P=0,6170
	Tunicamycin treatment	F (1, 16) = 74,08	P<0,0001 ***
	Interaction	F (1, 16) = 3,502	P=0,0797
bip	Genotype	F (1, 16) = 0,007929	P=0,9302
	Tunicamycin treatment	F (1, 16) = 30,31	P<0,0001 ***
	Interaction	F (1, 16) = 0,0002152	P=0,9885
hsp90b1	Genotype	F (1, 16) = 3,096	P=0,0976
	Tunicamycin treatment	F (1, 16) = 51,73	P<0,0001 ***
	Interaction	F (1, 16) = 3,403	P=0,0837
ire1	Genotype	F (1, 16) = 4,443	P=0,0512
	Tunicamycin treatment	F (1, 16) = 218,2	P<0,0001 ***
	Interaction	F (1, 16) = 5,350	P=0,0344 *
xbp1s	Genotype	F (1, 16) = 0,01421	P=0,9066
	Tunicamycin treatment	F (1, 16) = 31,72	P<0,0001 ***
	Interaction	F (1, 16) = 0,01421	P=0,9066
xbp1us	Genotype	F (1, 16) = 0,3365	P=0,5699
	Tunicamycin treatment	F (1, 16) = 29,26	P<0,0001 ***
	Interaction	F (1, 16) = 0,5956	P=0,4515
perk	Genotype	F (1, 16) = 4,275	P=0,0553
	Tunicamycin treatment	F (1, 16) = 61,18	P<0,0001 ***
	Interaction	F (1, 16) = 3,713	P=0,0720
eif2s1	Genotype	F (1, 13) = 0,002406	P=0,9616
	Tunicamycin treatment	F (1, 13) = 15,71	P=0,0016 **
	Interaction	F (1, 13) = 1,754	P=0,2082
atf4a	Genotype	F (1, 16) = 0,02127	P=0,8859

	Tunicamycin treatment	F (1, 16) = 18,30	P=0,0006 ***
	Interaction	F (1, 16) = 0,01598	P=0,9010
atf4b	Genotype	F (1, 16) = 3,545	P=0,0781
	Tunicamycin treatment	F (1, 16) = 31,66	P<0,0001 ***
	Interaction	F (1, 16) = 0,04306	P=0,8382
atf6	Genotype	F (1, 16) = 4,489	P=0,0501
	Tunicamycin treatment	F (1, 16) = 105,0	P<0,0001 ***
	Interaction	F (1, 16) = 4,620	P=0,0473 *
chop	Genotype	F (1, 11) = 1,601	P=0,2319
	Tunicamycin treatment	F (1, 11) = 27,50	P=0,0003 ***
	Interaction	F (1, 11) = 1,143	P=0,3080

**p < 0.05, **p < 0.01, ***p < 0.001*

Table S2. Two-way ANOVA statistical values for Figure 5.

	Factor	*F (DFn, DFd)*	*P value*
(B) Proteins	Ncs1 OE	F (1, 12) = 24,84	P=0,0003 ***
	Genotype	F (1, 12) = 0,02641	P=0,8736
	Interaction	F (1, 12) = 1,059	P=0,3236
(D) Distance	Ncs1 OE	F (1, 150) = 1,528	P=0,2184
	Genotype	F (1, 150) = 0,01625	P=0,8987
	Interaction	F (1, 150) = 0,6046	P=0,4380
(E) ON	Ncs1 OE	F (1, 150) = 1,708	P=0,1933
	Genotype	F (1, 150) = 2,256	P=0,1352
	Interaction	F (1, 150) = 1,805	P=0,1812
(F) OFF	Ncs1 OE	F (1, 150) = 7,986	P=0,0054 **
	Genotype	F (1, 150) = 7,434	P=0,0072 **
	Interaction	F (1, 150) = 6,419	P=0,0123 *

Abbreviation: Ncs1 OE, Ncs1 over-expression.

*$p < 0.05$, **$p < 0.01$, ***$p < 0.001$

Table S3. Two-way ANOVA statistical values for Figure 6.

	Factor	*F (DFn, DFd)*	*P value*
(B) Basal respiration	Ncs1 OE	F (1, 120) = 5,548	P=0,0201 *
	Genotype	F (1, 120) = 0,3201	P=0,5726
	Interaction	F (1, 120) = 2,743	P=0,1003
(C) ATP production	Ncs1 OE	F (1, 120) = 1,695	P=0,1954
	Genotype	F (1, 120) = 0,5904	P=0,4438
	Interaction	F (1, 120) = 0,1777	P=0,6741
(D) Maximal respiration	Ncs1 OE	F (1, 120) = 3,387	P=0,0682
	Genotype	F (1, 120) = 6,561	P=0,0117 *
	Interaction	F (1, 120) = 6,391	P=0,0128 *
(E) Proton leak	Ncs1 OE	F (1, 120) = 4,238	P=0,0417 *
	Genotype	F (1, 120) = 0,2717	P=0,6031
	Interaction	F (1, 120) = 6,468	P=0,0123 *
(F) Non mitochondrial	Ncs1 OE	F (1, 120) = 1,012	P=0,3165
respiration	Genotype	F (1, 120) = 1,891	P=0,1716
	Interaction	F (1, 120) = 3,352	P=0,0696

Abbreviation: Ncs1 OE, Ncs1 over-expression.

*$p < 0.05$

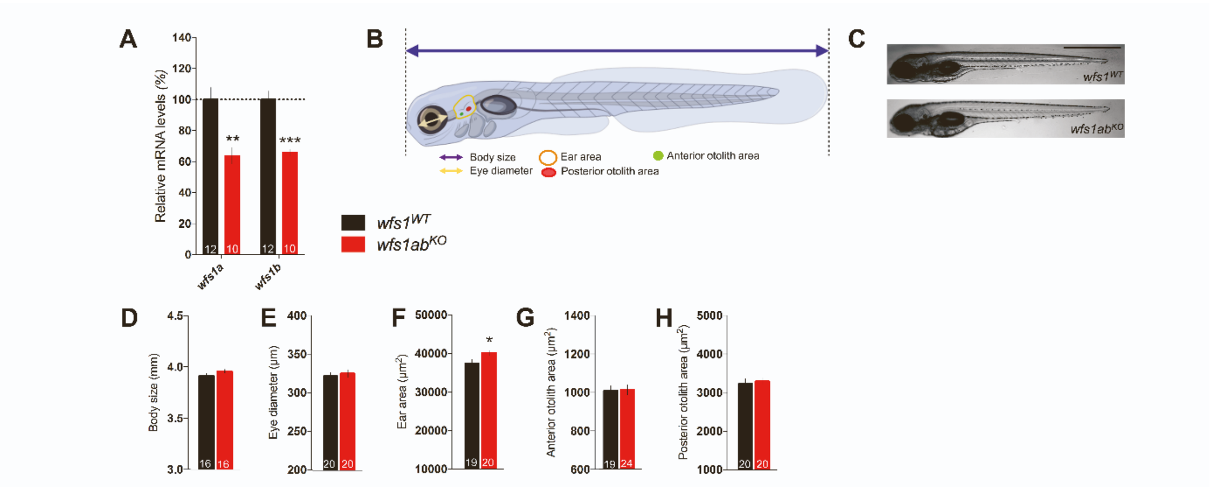

Figure S1. Characterization of *wfs1ab* mutant zebrafish line. (**A**) Relative *wfs1a* and *wfs1b* mRNA levels assessed by qPCR in *wfs1ab* zebrafish line, normalized against *zef1*α reference gene. (**B**) Schematic representation of the different measurements of the larva. (**C**) Representative images of wildtype and homozygous mutant *wfs1ab* larvae. Measurement of (**D**) body size, (**E**) eye diameter, (**F**) ear area, (**G**) anterior and (**H**) posterior otoliths area. Scale bars, 200 µm in D, 10 µm in the insets. Error bars represent mean ± SEM and the number of fish is indicated below the columns. *$p < 0.05$, **$p < 0.01$, ***$p < 0.001$; unpaired t-test.

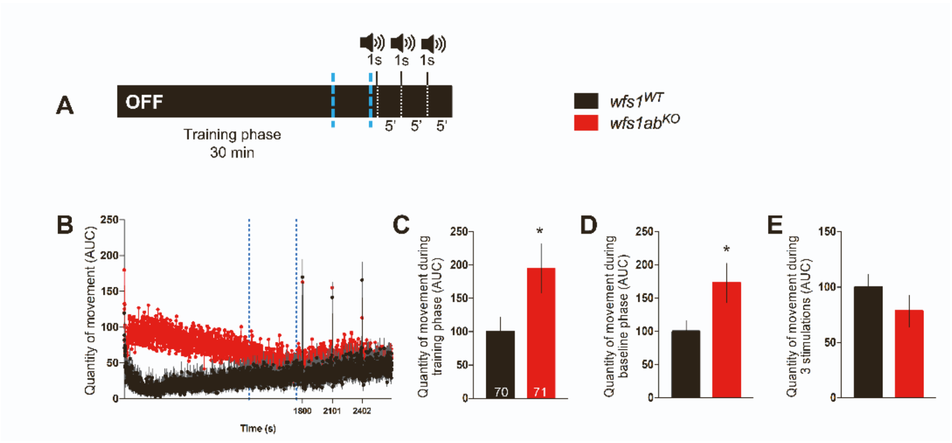

Figure S2. Analysis of the quantity of movement of *wfs1ab* mutant larvae during the noise cycle in the ASR test. (**A**) Sound protocol: the activity is measured for 45 min in a total dark (OFF) condition, with a training phase for 30 min silently, then 3 cycles of white sounds (90 dB) of 1 s each and 5 min interspersed. (**B**) Quantity of movement per second for the *wfs1ab*^{KO} larvae according to the sound protocol. Relative quantity of movement during: (**C**) the training phase [blue dotted lines in (**B**), between 21 and 29 min]; (**D**) the baseline phase, period of 2 min before each sound [the averaged 3 baseline phases]; (**E**) the 3 stimulations [the averaged sounds phases]. Activity was expressed as % of associated controls. Error bars represent ± SEM calculated from three replicas. The number of animals is indicated below the columns. *$p < 0.05$; unpaired t-test.

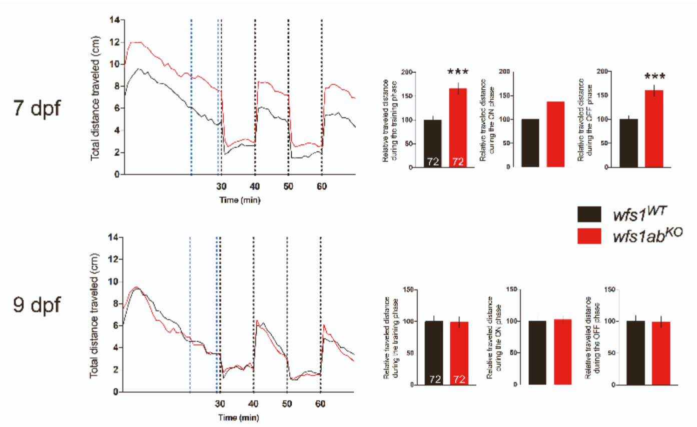

Figure S3. Behavioral analyses of 7-dpf and 9-dpf *wfs1ab*^{KO} zebrafish line. Analysis of the distance traveled by *wfs1ab*^{KO} larvae at 7-dpf (**A**) and 9-dpf (**E**) during the light/dark sequence in the VMR test: training is a light OFF period of 30 min, followed by two light ON and light OFF periods of 10 min each. Relative distance measured during: (**B**, **F**) the training phase over a 10-min period shown by blue dotted lines in (**A**, **E**); (**C**, **G**) the ON phases, averaged for ON1 and ON2; (**D**, **H**) the OFF phases, averaged for OFF1 and OFF2. Data show mean ± SEM, calculated from three replicas. The number of animals is indicated within the columns. ****p* < *0.001;* unpaired t-test.

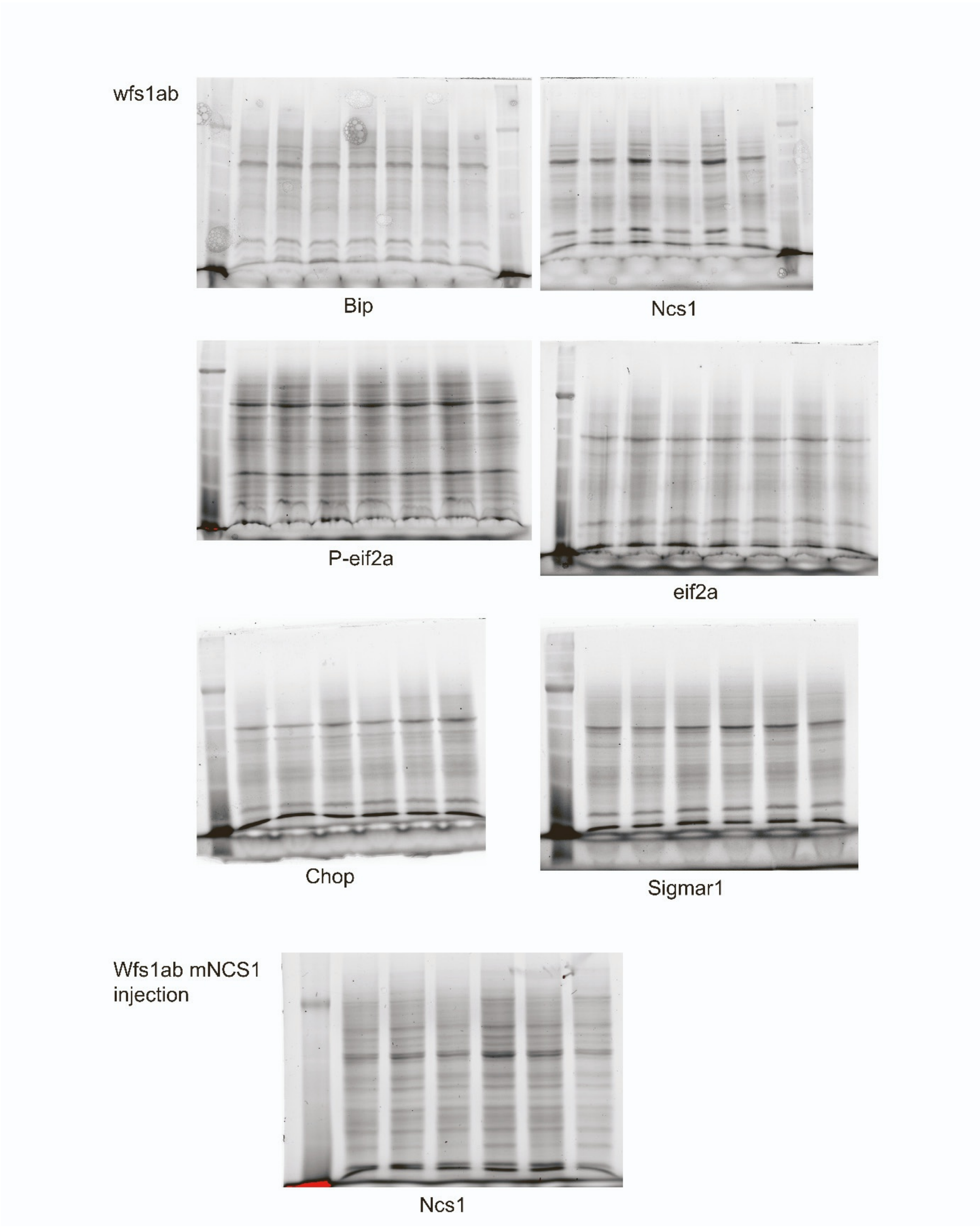

Figure S4. Stain Free for Western blots from Figure 3.
